# Neural selectivity for social interactions in the infant brain

**DOI:** 10.1016/j.isci.2026.116287

**Published:** 2026-06-08

**Authors:** Manuel Mello, Emilie Serraille, Jean-Rémy Hochmann, Liuba Papeo

**Affiliations:** 1 Institut des Sciences Cognitives—Marc Jeannerod, UMR5229, Centre National de La Recherche Scientifique (CNRS) and Université Claude Bernard Lyon 1, 67 Bd. Pinel, 69675 Bron, France

**Keywords:** health sciences, medicine, neurology, pediatrics, human physiology, natural sciences, biological sciences, neuroscience, developmental neuroscience, cognitive neuroscience

## Abstract

Social cognition develops in the second year of life. Yet even in the first months, infants show sophisticated representation of social interactions. The neural structures supporting these early capacities remain unknown. Here, using functional near-infrared spectroscopy, we show that, like adults, preverbal infants exhibit selective increase in the activity of posterior superior-temporal and temporo-parietal regions when viewing individuals facing and moving toward (vs. away from) each other, as if interacting. No reliable difference emerged between 6- and 10-month-olds, although neural effects were more widespread in the older group, who also showed consistently longer looking times toward interacting dyads. Neural effects of social interactions were spatially distinct from effects of body motion perception, indicating separate mechanisms for perceiving biological agents and interacting agents. The early tuning to visuo-spatial cues of social engagement documented here may orient infants’ attention toward social interactions, thereby facilitating the discovery and learning of human social relationships.

## Highlights

• Neural selectivity for stimuli depicting social interaction emerges by 6 months of age
• Posterior temporo-parieto-occipital areas respond more to interacting vs. noninteracting dyad
• By 10 months infants show increased attention to social interactions
• Tuning to social interaction may support the early stages of social learning

## Graphical abstract

**Figure:**
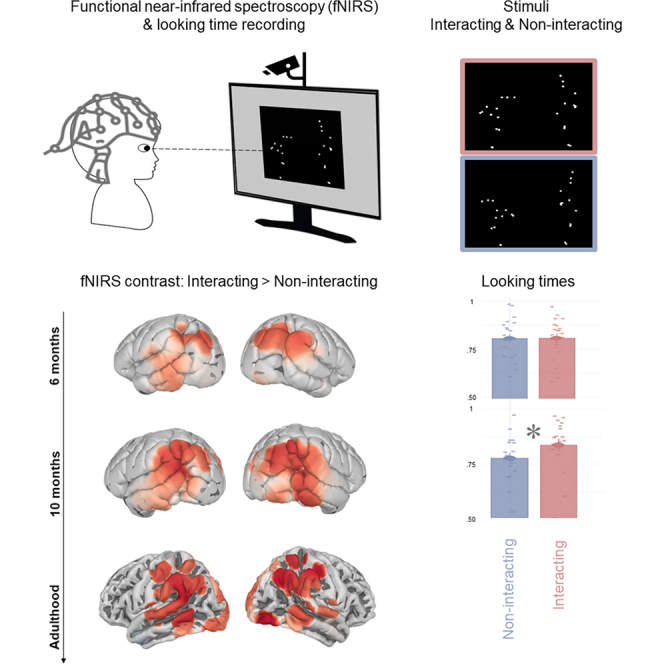

## Introduction

How humans represent social relationships is a question at the heart of social sciences. Theoretical and empirical work offers numerous attempts to characterize how human societies are organized and how this organization reflects the categorization of social interactions by the human mind.^1^^,^^2^^,^^3^^,^^4^ A model that explains how humans represent social relationships should be also—and first—concerned with the question of how one recognizes that there is a social relationship, or that two social agents are connected.^5^^,^^6^ Answering this question can also critically help to explain how representations of social relationships first emerge in infancy; particularly, whether there are specific signals that facilitate or bias the representation of two agents as connected.

Investigating the processing of visual events depicting physical or communicative exchanges (i.e., social interactions where at least one agent acts to affect the state of at least another agent) offers an opportunity to address this question. It can shed light onto the fundamental process by which infants, by observing others, recognize social interactions and, from there, learn about social norms, social roles, hierarchies, intimacy, and other aspects of social living.

In the second year of life, infants manifest sophisticated abilities for evaluating complex social events and making inferences about dominance, pro-sociality, and intimacy.^6^^,^^7^^,^^8^^,^^9^^,^^10^^,^^11^^,^^12^ However, already by 6 months, infants can represent aspects of social interactions such as affiliation, and social roles such as helper/hinderer^13^^,^^14^ (but see Lucca et al.^15^) or agent/patient,^16^ based on signals such as the mutual perceptual accessibility of two individuals,^17^^,^^18^ body postures and positioning,^16^ motion synchrony,^19^ and coordination^14^ (see Thomas^6^). What is the neural mechanism behind such early social-perception abilities?

Neuroimaging research investigating the processing of social interactions in the human brain has highlighted a key role of a posterior superior temporal sulcus (pSTS) region in the perception of social interactions,^20^^,^^21^^,^^22^^,^^23^^,^^24^^,^^25^^,^^26^^,^^27^ separate from effects of other forms of social processing such as face, body, and motion perception in nearby visual areas, theory-of-mind processing in the nearby temporoparietal junction (TPJ),^22^^,^^27^^,^^28^^,^^29^ and the processing of social information in medial prefrontal cortex (mPFC).^30^^,^^31^ These results have encouraged the thinking that perceptual representations of social interactions arise in pSTS, integrating inputs from upstream occipitotemporal regions that encode visual relational information—such as, for example, the spatial positioning of two individuals.^24^^,^^28^^,^^32^^,^^33^^,^^34^^,^^35^^,^^36^

Functional MRI (fMRI) studies have shown pSTS activity in response to social interactions, in 6-year olds and older children.^37^^,^^38^ Moreover, a reanalysis of fMRI data from 3- to 8-year-old children watching movies,^39^ found that, from 3 years, the presence of a social interaction predicted neural activity in a widespread network encompassing the superior temporal gyrus and TPJ.^40^ But, 3 years is a relatively late stage in social and cognitive development. Farris et al.^41^ were the first to record neural activity in 6- to 13-month olds while they watched video-clips of people interacting face-to-face (vs. non-interacting people). Using functional near-infrared spectroscopy (fNIRS), they reported stronger activity in the superior temporal cortex, in addition to mPFC activity, for interacting people.^30^^,^^31^ However, the fNIRS channel layout was not designed to record activity in posterior visual aspects of STS and occipitotemporal cortex, thus leaving open the question of whether visual areas in infants, like in adults, show selectivity for stimuli carrying cues of social engagement.

Here, we asked whether selectivity for social interaction in pSTS (i.e., stronger activity for interacting vs. non-interacting people) is present early in life, thus providing a candidate mechanism to support infants’ early processing of social interaction. The present study is the first to measure neural responses to social interactions specifically targeting posterior cortical areas in infants as young as 6 and 10 months. Neural activity was recorded with fNIRS while infants watched video-clips of interacting (vs. non-interaction) individuals, where interaction (or lack thereof) was conveyed by visuo-spatial cues such as body orientation and movement direction (toward vs. away from one another). Bodies were presented as point-light displays (PLDs), with light points on a dark background marking the main bodily joints (e.g., wrists, elbows, and knees).^42^ This format elicits a vivid percept of body motion while eliminating other visual cues (e.g., color, texture, facial features, and expressions).^43^^,^^44^ Before testing infants, we performed the same measurement in a group of adults, to verify that with fNIRS we could obtain reliable effects, compatible with those found with fMRI during visual perception of social interactions.^21^^,^^22^^,^^23^^,^^26^^,^^27^^,^^28^^,^^32^^,^^33^ To preview, the results provide the first evidence of early-developing selectivity for social interaction in brain areas identified as key components of a neural *pathway* for social-interaction perception.^25^^,^^35^^,^^45^

## Results

### Experiment 1

**Figure 1:**
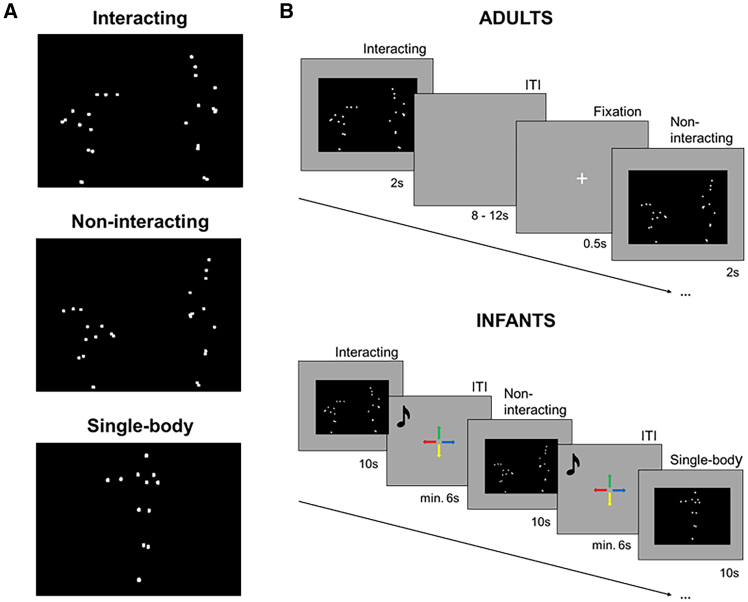
Illustration of stimuli and experimental designs in experiments 1 and 2 (A) Stimuli. Snapshots of PLDs of interacting and non-interacting dyads (experiments 1 and 2) and single-body movements (experiment 2). (B) Extract of the experiment. Sequence of events in the slow-event related designs implemented in experiments 1 and 2. In experiment 1 (*N* = 20 adults), participants were presented with 2-s video-clips of interacting or non-interacting dyads. Trials were interleaved with 8–12 s intertrial interval (ITI, jittered). In experiment 2, infants (20 6-month olds and 21 10-month olds) were presented with 10-s video-clips of interacting, non-interacting, and single-body PLDs, presented in random order and interleaved with an ITI of minimum 6 s, with two 3-s bell sounds.

**Figure 2:**
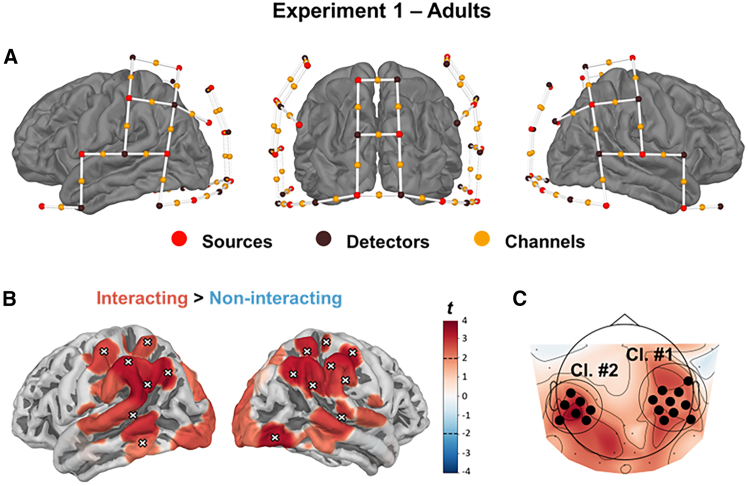
Results of experiment 1 (A) 3D illustration of the fNIRS channel layout used in experiment 1 (adults). (B) Distribution of the response for the [interacting > non-interacting] contrast in the channel-wise analysis. White Xs indicate the location of channels showing a significant effect of interacting > non-interacting stimuli. (C) Results of the cluster-based permutation test revealing two posterior clusters (Cl. #1 right and #2 left) at the intersection of the occipital, temporal, and parietal cortex (see Table S3 for channel specifics).

Human neuroimaging research has revealed selectivity to social interaction in a *pathway* of visual areas centered in pSTS. Evidence primarily relies on fMRI data in human adults^20^^,^^21^^,^^22^^,^^23^^,^^24^^,^^25^^,^^26^^,^^27^^,^^28^^,^^29^^,^^32^^,^^33^^,^^34^ and, to a lesser extent, children.^37^^,^^38^^,^^39^^,^^40^ Here, we assessed whether fNIRS could reliably capture the effects observed with fMRI in adults. Twenty healthy adults were presented with 2-s video-clips featuring two human bodies, in the form of PLDs, performing familiar movements either face-to-face or back-to-back (Figure 1), interpersonal orientations that yield representation of interacting or non-interacting people, respectively.^18^^,^^46^^,^^47^ During stimulus presentation, oxyhemoglobin (HbO) concentration, a correlate of cortical activity^48^ was measured with a 37-channel fNIRS with a channel layout optimized to record from occipital, posterior temporal, and posterior parietal cortices (Figure 2A; Tables S1 and S2). By-channel, by-condition HbO concentration was extracted for each participant and analyzed offline at the group level. We report the same analyses on de-oxyhemoglobin (HbR) and total (HbT) concentrations as supplemental information.

The fNIRS map resulting from the HbO contrast interacting > non-interacting stimuli was subjected to a no-intercept mixed linear model with channel as fixed effect and participant as random factor. This analysis revealed stronger response to interacting (vs. non-interacting) dyads in 16 of the 37 channels, distributed over occipitoparietal and posterior temporoparietal regions (all *t*s > 1.98, all *p*s < 0.05) (Figure 2B; Table 1). For 14 of these channels, Bayesian hypothesis testing showed anecdotal to substantial evidence in favor of H_1_ (higher response for interacting dyads; 1.02 < BF_s_ < 6; Table 1; see supplemental information, pp. 22–23, for a full report of Bayesian statistics). No significant effect was found for the opposite contrast, non-interacting > interacting stimuli.

**Table 1:** Channels showing a significant interacting > non-interacting effect in the channel-wise analysis of experiment 1 (adults)

Channel	AAL	MNI coordinates (*x*, *y*, *z*)	Statistics
POO10h-PPO10h	CERCRU1 (R) (49%) IOG (R) (21%)	41, −79, −23	*est.* = 1.26 ± 0.42, *t*_(182.69)_ = 2.99, *p* = 0.003, BF_10_ = 4.39
CPP6h-PPO6h	ANG (R) (47%) MOG (R) (42%)	48, −71, 31	*est.* = 1.18 ± 0.42, *t*_(182.69)_ = 2.81, *p* = 0.005, BF_10_ = 3.37
CPP6h-TPP8h	MTG (R) (45%) ANG (R) (36%)	58, −58, 22	*est.* = 1.03 ± 0.42, *t*_(182.69)_ = 2.45, *p* = 0.01, BF_10_ = 1.55
CPP6h-CPP4h	ANG (R) (63%) IPG (R) (26%)	47, −62, 47	*est.* = 1.13 ± 0.42, *t*_(182.69)_ = 2.7, *p* = 0.007, BF_10_ = 2.42
CPP6h-CCP6h	SMG (R) (42%) IPG (R) (32%) ANG (R) (24%)	58, −47, 38	*est.* = 0.96 ± 0.42, *t*_(182.69)_ = 2.84, *p* = 0.02, BF_10_ = 1.27
CCP4h-CPP4h	SPG (R) (50%)	39, −50, 60	*est.* = 1.09 ± 0.42, *t*_(182.69)_ = 2.6, *p* = 0.01, BF_10_ = 1.99
CCP4h-CCP6h	SPG (R) (39%) SMG (R) (28%)	53, −36, 51	*est.* = 1.31 ± 0.42, *t*_(182.69)_ = 3.12, *p* = 0.002, BF_10_ = 4.84
TTP8h-TPP8h	MTG (R) (76%) STG (R) (21%)	65, −44, 6	*est.* = 0.97 ± 0.42, *t*_(182.69)_ = 2.3, *p* = 0.02, BF_10_ = 1.32
TTP8h-CCP6h	SMG (R) (49%) STG (R) (43%)	65, −33, 22	*est.* = 0.83 ± 0.42, *t*_(182.69)_ = 1.98, *p* = 0.04, BF_10_ = 0.96
CPP3h-CPP5h	ANG (L) (50%) IPG (L) (40%)	−46, −62, 46	*est.* = 0.86 ± 0.42, *t*_(182.69)_ = 2.04, *p* = 0.04, BF_10_ = 1.03
PPO5h-CPP5h	ANG (L) (58%) MOG (L) (31%)	−47, −71, 30	*est.* = 1.25 ± 0.42, *t*_(182.69)_ = 2.98, *p* = 0.003, BF_10_ = 4.91
TPP7h-CPP5h	MTG (L) (48%) ANG (L) (29%)	−57, −58, 21	*est.* = 1.28 ± 0.42, *t*_(182.69)_ = 3.04, *p* = 0.002, BF_10_ = 6.05
TPP7h-TPP9h	MTG (L) (60%) ITG (L) (35%)	−64, −51, −9	*est.* = 1.01 ± 0.42, *t*_(182.69)_ = 2.41, *p* = 0.01, BF_10_ = 1.48
TPP7h-TTP7h	MTG (L) (83%)	−65, −44, 4	*est.* = 0.84 ± 0.42, *t*_(182.69)_ = 1.99, *p* = 0.04, BF_10_ = 0.96
CCP5h-CPP5h	IPG (L) (52%) SMG (L) (34%)	−57, −47, 37	*est.* = 1.26 ± 0.42, *t*_(182.69)_ = 3.01, *p* = 0.002, BF_10_ = 3.88
CCP5h-CCP3h	IPG (L) (52%)	−53, −34, 51	*est.* = 0.88 ± 0.42, *t*_(182.69)_ = 2.09, *p* = 0.03, BF_10_ = 1.03

*Note*. “Channel” labels denote landmarks of the 10-5 electrode positioning system.^49^ AAL labels reflect probabilistic mapping of underlying cortical areas (specificity in parentheses) as calculated in Zimeo Morais et al.^50^ CERCRU1, Crus I of cerebellar hemisphere; I/MOG, inferior/middle occipital gyrus; I/M/STG, inferior/middle/superior temporal gyrus; ANG, angular gyrus; I/SPG, inferior/superior parietal gyrus; SMG, supramarginal gyrus.

A one-sample cluster-based permutation on the selectivity values [interacting – non-interacting] was used to determine, in a data-driven way, channels that clustered together. This analysis revealed two significant clusters: cluster #1 (*p* = 0.01) encompassed 10 channels over the right occipitotemporal, occipitoparietal, and temporoparietal cortex; cluster #2 (*p* = 0.02) consisted of seven channels over the left occipitotemporal, occipitoparietal, and temporoparietal cortex (Figure 2C; see Table S3 for channels specifics). The remaining 20 channels, covering most of the occipital cortex and anterior sections of the temporal and parietal cortex, did not show an effect.

These analyses confirmed the visual selectivity for face-to-face dyads in posterior middle occipital and temporoparietal cortex. In line with the current literature,^35^ we propose that this univariate effect arises from the representation of the relationship between two individuals cued by face-toward interpersonal orientation and movement direction. Importantly for the purposes of this study, these results demonstrated that fNIRS can reliably replicate neural effects of social-interaction perception, previously observed with fMRI in adults and children.

### Experiment 2

**Figure 3:**
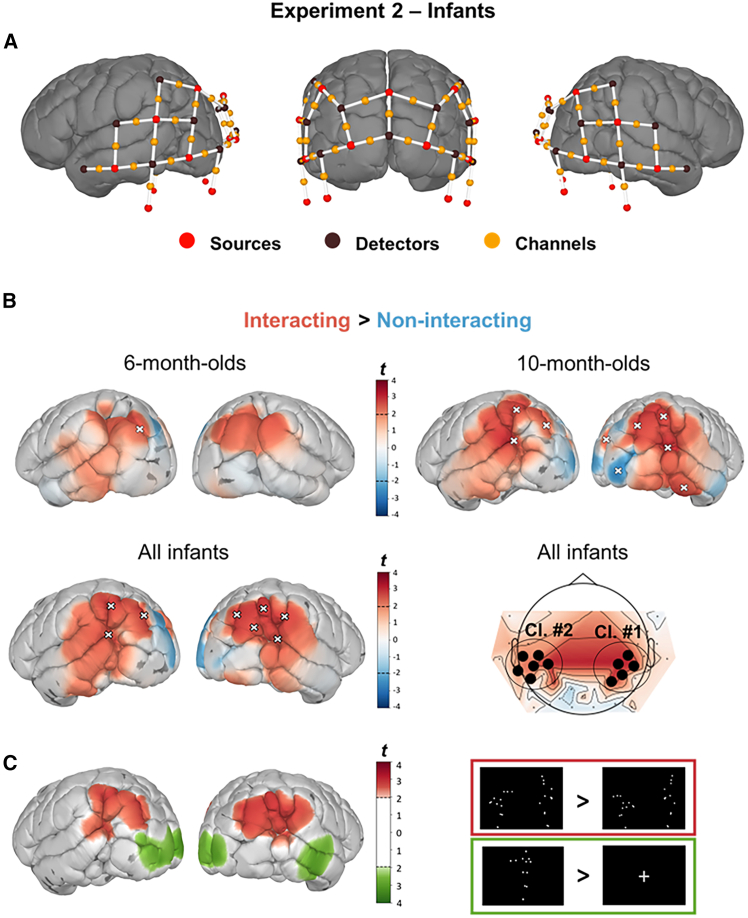
Results of experiment 2 (A) 3D illustration of the fNIRS channel layout used in experiment 2 (6- and 10-month old infants). (B) Distribution of the response for the contrast [interacting > non-interacting] in the channel-wise analysis in 6-month olds, 10-month olds, and all infants together (see supplemental information, pp. 11–14, for a details on separate analyses of 6- and 10-month olds). White Xs indicate the location of channels showing a significant effect. The bottom right image displays the results of the cluster-based permutation test on all infants revealing two clusters, respectively in right occipitoparietal and temporoparietal cortex and left occipitotemporal and temporoparietal cortex (see Table S8 for channel specifics). (C) Distribution of the significant social-interaction effect (in red) and of the significant response to single-body motion (vs. baseline; in green).

Two groups of infants (20 6-month olds and 21 10-month olds) were presented with PLDs depicting interacting (face-to-face) and non-interacting (back-to-back) dyads, similar to those of experiment 1, but of longer duration (10 s each). In addition, infants saw a set of 10-s videos showing PLDs featuring a single moving body (see “STAR Methods”). The conditions with dyads were included to test neural effects of social-interaction perception in infants. The condition with single bodies was included to assess whether, like in adults,^22^^,^^27^^,^^28^^,^^29^ in infants, social-interaction perception is spatially segregated from other forms of social processing such as person (body/bodily motion) perception. Infants watched the videos while cortical activity was recorded with a 39-channel fNIRS layout covering occipitotemporal and temporoparietal cortices (Figure 3A; Tables S6 and S7).

A mixed linear model assessing the effects of age and channel on neural selectivity for social interaction (differential HbO response for interacting > non-interacting) in infants, revealed a main effect of channel, *F*_(39,742)_ = 1.57, *p* = 0.01, BF_10_ = 13.47, but no effect of Age, *F*_(1,39)_ = 0.09, *p* = 0.76, BF_10_ = 0.82, or interaction, *F*_(38,1482)_ = 0.58, *p* = 0.98, BF_10_ = 0.13. The difference between interacting and non-interacting conditions was significant in eight channels (all *t*s ≥ |1.96|, all *p*s ≤ 0.05). In these channels, covering occipitoparietal and temporoparietal areas bilaterally, the response was higher for interacting dyads (Figure 3B; Table 2). BF_10_s calculated for the parameters of significant channels (main effect of channel) provided anecdotal to substantial evidence in favor of H_1_ (higher response for interacting dyads; 1.02 < BF_10s_ < 6; see Table 2; see also Method S1, pp. 23–25 for a full report of Bayesian statistics).

**Table 2:** Channels showing a significant interacting > non-interacting effect in the channel-wise analysis of experiment 2

Channel	AAL	Coordinates (*x*, *y*, *z*)	Statistics
P4-PO4	MOG (R) (61%) ANG (R) (26%)	32, −75, 36	*est.* = 1.71 ± 0.62, *t*_(653.85)_ = 2.77, *p* = 0.006, BF_10_ = 2.31
P4-P6	ANG (R) (78%	45, −65, 32	*est.* = 1.21 ± 0.62, *t*_(653.85)_ = 1.96, *p* = 0.05, BF_10_ = 1.07
P4-CP4	IPG (R) (63%) ANG (R) (32%)	44, −54, 47	*est.* = 1.63 ± 0.62, *t*_(653.85)_ = 2.64, *p* = 0.008, BF_10_ = 2.39
CP6-P6	MTG (R) (37%) STG (R) (31%)	55, −52, 22	*est.* = 1.5 ± 0.62, *t*_(653.85)_ = 2.43, *p* = 0.01, BF_10_ = 1.54
CP6-CP4	SMG (R) (42%) IPG (R) (42%)	56, −42, 36	*est.* = 1.48 ± 0.62, *t*_(653.85)_ = 2.39, *p* = 0.01, BF_10_ = 1.89
CP5-P5	MTG (L) (42%) STG (L) (34%)	−57, −49, 20	*est.* = 1.94 ± 0.62, *t*_(653.85)_ = 2.25, *p* = 0.02, BF_10_ = 1.68
P3-CP3	IPG (L) (51%) ANG (L) (39%)	−49, −52, 44	*est.* = 1.58 ± 0.62, *t*_(653.85)_ = 2.56, *p* = 0.01, BF_10_ = 2.01
P3-PO3	MOG (L) (51%) ANG (L) (32%)	−36, −73, 34	*est.* = 1.76 ± 0.62, *t*_(653.85)_ = 2.85, *p* = 0.005, BF_10_ = 2.71

*Note.* “Channel” labels denote landmarks of the 10-10 electrode positioning system.^49^ AAL labels reflect probabilistic mapping of underlying cortical areas (specificity in parentheses) as calculated in Fu and Richards.^51^ MOG, middle occipital gyrus; I/M/STG, inferior/middle/superior temporal gyrus; ANG, angular gyrus; IPG, inferior parietal gyrus; SMG, supramarginal gyrus.

A data-driven approach using a cluster-mass permutation test on the selectivity values [interacting – non-interacting], revealed two significant clusters: cluster #1 (*p* = 0.02) encompassed five channels over the right occipito-parietal and temporoparietal cortex; cluster #2 (*p* = 0.03) encompassed six channels over the left occipitotemporal and temporoparietal cortex (Figure 3B, bottom right image; see Table S8 for channel specifics). The remaining 28 channels, covering most of the occipital cortex and anterior sections of the temporal and parietal cortex, did not yield any significant cluster. A comparison of the response of the two age groups within each cluster revealed no significant age effect (cluster #1: *F*_(1,39)_ = 0.02, *p* = 0.89; cluster #2: *F*_(1,39)_ = 0.19, *p* = 0.66). This was confirmed by the BF_10_s calculated for these comparisons, which indicated anecdotal evidence in favor of H_0_ of no age difference (cluster #1: BF_10_ = 0.98; cluster #2: BF_10_ = 0.68). Thus, our findings show that, like adults, preverbal infants displayed neural selectivity for social interaction in posterior occipitotemporal and temporoparietal regions. The analysis for each age group separately is reported as supplemental information.

Further analyses were conducted to examine whether the perception of social interaction elicited neural effects that could be spatially segregated from those evoked by the perception of single body motion. First, using mixed-effects modeling, we assessed the absolute hemodynamic response to body motion by comparing the estimated by-channel responses against baseline (zero). This analysis revealed that perception of body motion reliably activated six channels over the lateral and middle occipital cortex, bilaterally, and the right middle/superior temporal cortex (Figure 3C; Table 3). An effect of body motion perception in the anterior aspects of the temporal cortex (channels: T8-TP8, T8-C6, CP6-TP8) has already been observed using fNIRS in infants.^52^^,^^53^ Here we showed that this effect is segregated from the effect of social-interaction perception. In fact, a conjunction analysis on the maps [interacting > non-interacting] and [single-body > baseline] revealed no overlap between the two effects (Figure 3C; see also supplemental information, pp. 11–14).

**Table 3:** Channels showing a significant effect of single-body motion perception (vs. baseline) in the channel-wise analysis of experiment 2

Channel	AAL	Coordinates (*x*, *y*, *z*)	Statistics
O2-Oz	MOG (R) (51%) CAL (R) (26%)	10, −91, 9	*est.* = 1.13 ± 0.38, *t*_(479.30)_ = 3, *p* = 0.003, BF_10_ = 13.39
CP6-TP8	MTG (R) (78%)	60, −39, 7	*est.* = 0.82 ± 0.38, *t*_(479.30)_ = 2.18, *p* = 0.03, BF_10_ = 2.44
T8-TP8	MTG (R) (71%) ITG (R) (29%)	61, −29, −11	*est.* = 1.52 ± 0.38, *t*_(479.30)_ = 4.04, *p* < 0.001, BF_10_ = 109.16
T8-C6	MTG (R) (48%) STG (R) (21%)	62, −15, 3	*est.* = 0.88 ± 0.38, *t*_(479.30)_ = 2.35, *p* = 0.01, BF_10_ = 3.19
O1-OZ	CAL (L) (31%)	−13, −90, 8	*est.* = 0.81 ± 0.38, *t*_(479.30)_ = 2.14, *p* = 0.03, BF_10_ = 2.82
O1-PO7	MOG (L) (49%) IOG (L) (44%)	−33, −81, 6	*est.* = 1.16 ± 0.38, *t*_(479.30)_ = 3.08, *p* = 0.002, BF_10_ = 13.49

*Note.* CAL, calcarine fissure and surrounding cortex; I/MOG, inferior/middle occipital gyrus; I/M/STG, inferior/middle/superior temporal gyrus. AAL labels represent probabilistic mapping of underlying cortical areas (specificity in parentheses).

Additional analyses were carried out to assess the relationship between pSTS activity and attention. Since aspects of pSTS have been implicated in the attention network,^54^^,^^55^ we asked to what extent the present pSTS effects reflected the variance in the attention to the different types of stimuli. To test this, we extracted the looking times toward each stimulus from the videos of infants, recorded during the fNIRS section (for details, see “Looking time analyses”, pp. 20–22 of supplemental information). An ANOVA with age (6 and 10 months) as between-subjects factor and condition (interacting, non-interacting, single) as within-subjects factor revealed a main effect of condition, *F*_(2)_ = 3.58, *p* = 0.03, no an effect of age, *F*_(1)_ = 0.51, *p* = 0.48, and a significant interaction between the two, *F*_(2)_ = 4, *p* = 0.02. The interaction reflected the fact that 10-month olds looked longer at interacting bodies and single bodies, relative to non-interacting bodies (interacting: 0.84 ± 0.12; non-interacting: 0.79 ± 0.1; single-body: 0.87 ± 0.13), whereas 6-month olds showed no difference between conditions (interacting: 0.81 ± 0.11 *SD*; non-interacting: 0.81 ± 0.11; single-body: 0.81 ± 0.11). Pairwise comparisons (Tukey-corrected) confirmed that 10-month olds looked longer at interacting vs. non-interacting dyads (est. = 0.06 ± 0.02, *t* = 2.44, *p* = 0.04) and at single-bodies vs. non-interacting dyads (est. = 0.09 ± 0.02, *t* = 3.85, *p* < 0.001) (all other comparisons: *t*s < 1.42, *p*s > 0.34) (Figure S8).

Next, we tested whether the variation in looking times, taken as an index of attention, could fully account for the pSTS response to interacting and non-interacting dyads. To this end, looking times were entered as a covariate in two separate linear mixed models, one for the left pSTS (channels CP5-P5) and the other for the right pSTS (CP6-P6), with Age as between-subjects factor and Condition (interacting, non-interacting) as within-subjects factor. Results showed that, in both left and right pSTS, the effect of social interaction persisted after accounting for the variance in looking times. In particular, in left pSTS, an effect of Condition was found, *F*_(1,36)_ = 6.59, *p* = 0.01, with higher response to interacting than non-interacting dyads (est. = 1.87 ± 0.71, *t* = 2.64, *p* = 0.01) and no modulation of looking times, *F*_(1,54)_ = 1.51, *p* = 0.22. Likewise, in right pSTS, we found an effect of condition, *F*_(1,36)_ = 9.32, *p* = 0.004, with higher response to interacting than non-interacting dyads (est. = 1.73 ± 0.56, *t* = 3.01, *p* = 0.004), and no modulation of looking times, *F*_(1,54)_ = 0.76, *p* = 0.39 (all other main effects and interactions: *F*s < 1.68, *p*s > 0.2).

In summary, attention to stimuli alone—at least the attention component captured by looking times—is insufficient to explain pSTS activation, thereby implying a critical role of stimulus category (interacting/non-interacting dyads).

## Discussion

Already in the first months of life, infants exhibit a rich representation of third-party social interactions: from body postures, spatial positioning, and motion patterns of social agents, they represent affiliation^19^ and social roles (e.g., helper/hinderer or agent/patient^13^^,^^14^^,^^16^; but see Lucca et al.^15^). Here, we asked which neural mechanisms may support the processing of social interaction in early infancy.

Human neuroimaging research has identified a *pathway* of visual areas centered in pSTS, in which representations of social interaction may be constructed from visuo-spatial relational information such as interpersonal distance, orientation, and motion direction.^21^^,^^24^^,^^35^^,^^36^ We asked whether this neural mechanism, revealed by a selectivity for stimuli carrying visuo-spatial cues of social engagement, is available early in infancy, as a possible substrate for early social-interaction perception. To test this, we manipulated interpersonal orientation (face-to-face vs. back-to-back) to cue interacting or non-interacting dyads, and used fNIRS to record neural activity in 6- and 10-month-old infants—as well as in adults.

fNIRS results in adults provided an important cross-methodology replication of previous fMRI results,^20^^,^^21^^,^^22^^,^^23^^,^^24^^,^^25^^,^^26^^,^^27^^,^^28^^,^^29^^,^^32^^,^^33^^,^^34^^,^^35^ showing increased activity in occipitotemporal and temporoparietal cortex, including pSTS, in response to interacting (vs. non-interacting) individuals. This univariate effect has been interpreted as reflecting the representation of emergent properties that arise when individuals are perceived as connected or interacting by virtue of their spatial relationship.^36^^,^^56^^,^^57^

The effect observed in young infants was strikingly similar to the effect found in adults. There was no statistically reliable difference between 6- and 10-month olds. However, by visual inspection, the effect in 6-month olds was less widespread than in 10-month olds (Figure 3B and Method S1), suggesting that selectivity for social interactions in visual cortex emerges early—by 6 months of age—and continues to develop in degree, at least over the following months, and likely over subsequent years (see Sapey-Triomphe et al.,^37^ Im et al.,^40^^,^ and Farris et al.^41^). Given the high variability of the hemodynamic response in infants, especially the younger ones,^58^ we cannot rule out that a larger sample size might reveal age-related differences, although Bayesian analyses in the present study provides evidence supporting the absence of such differences.

Interpreted in the framework of the research on adults, these effects suggest that, in infants, face-toward body orientation and movement direction trigger further neural processing that signal the emergence of a representation of social interaction from body and motion perception. In particular, these findings support a role of pSTS and occipitotemporal regions in processing visual relational cues—such as interpersonal orientation and approach—that are reliably associated with social interaction.^21^^,^^25^^,^^35^ A future direction will be to disentangle, in both infants and adults, the contribution of these two relational cues to the formation of social interaction representations. Moreover, the effect of social interaction in occipitotemporal and temporoparietal cortex was spatially segregated from the effect of perceiving just body motion. This demonstrates that the processing of third-party social interactions is a specific early-developing component of social processing, separate from other forms of social processing like body and biological motion perception.

Finally, the looking-time analysis clarified that, while pSTS may be sensitive to attention,^54^^,^^55^ attention alone cannot account for the effect of interacting (vs. non-interacting) dyads. First, 10-month olds—but not 6-month olds—looked longer at interacting than non-interacting dyads. This finding is in-line with extant work showing that visual preference for face-to-face individuals emerges progressively only after 6 months of age.^9^^,^^10^^,^^11^^,^^12^^,^^13^^,^^14^^,^^15^^,^^16^^,^^17^^,^^18^ However, the effect of social interaction (higher response to interacting vs. non-interacting dyads) in pSTS persisted after accounting for the variance in looking times. Second, 10-month olds looked at single bodies as much as at interacting dyads; yet, there was no increase of pSTS activity in response to single bodies (Figure 3). Third, like 10-month olds, 6-months olds showed pSTS selectivity for interacting dyads; yet, they did not look longer at interacting than non-interacting dyads.

In sum, by 6 months, occipitotemporal areas are biased to respond preferentially to social interactions—or at least to complex visual structures carrying the proxemics of social interaction (i.e., spatial proximity, facingness, and approaching behavior). This tuning may gradually increase attention toward social interactions facilitating the discovery and learning of human social relationships. But, how does the neural selectivity to social interactions emerge?

The selectivity to social interactions in visual areas could be a purely visual phenomenon. In particular, it may reflect the functional properties of areas along the third visual pathway^25^ that respond to (or receive projections from early visual areas that respond to) recurrent features in social interactions, and/or the existence of an innate visual template of two face-to-face bodies (see Powel et al.^59^ for a similar discussion about face perception). Innateness is the signature of evolutionarily ancient functions, shared with other species along the phylogeny. A recent study^60^ has reported a preference for face-to-face dyads in newly hatched chicks. In particular, the study showed that, at birth, domestic chicks spontaneously move toward and spend more time around a screen showing two hens moving toward one another vs. two hens moving back-to-back. Innate preference for social stimuli in domestic chickens likely relies on subcortical visual mechanisms that have also been proposed to explain the visual sensitivity to faces in human newborns.^59^^,^^61^^,^^62^ Thus, the preference for face-to-face dyads in newly hatched chicks^60^ raises the possibility that selectivity for face-to-face dyads in human visual areas reflects a biologically determined mechanism encoded in subcortical and cortical visual structures. If so, neural selectivity for facingness may emerge even before 6 months of age—or possibly at birth—and may involve brain structures not adequately captured by the present methodology. Research in younger infants and newborns using higher-resolution methods, as they become more available,^63^^,^^64^ represents an exciting avenue for future investigation.

The selectivity for face-to-face people may emerge gradually as infants accumulate experience with watching third-party social interactions, and/or learn to generalize social cues relevant for first-person social interactions (e.g., gaze toward oneself) to interactions between others (e.g., gaze toward another) (see Thiele et al.^9^ and Goupil et al.^18^). Importantly, innateness-based and experience-based hypotheses are not mutually exclusive as interactions between innate dispositions and postnatal experience have been proposed to account for the development of face^65^ and biological motion perception.^66^ Gradual developmental changes may also reflect the progressive involvement and maturation of cortical structures that come to support mechanisms initially—albeit more coarsely—subserved by subcortical structures.^61^^,^^67^ In addition, they may reflect the development of long-range connectivity between posterior areas encoding the visual structure of social interactions, and prefrontal areas, implicated in the representation of the social value of stimuli.^59^^,^^68^^,^^69^ In young infants, the mPFC responds to a range of social signals^30^ including face-to-face social interactions^41^ and, in children and adolescents, this region is functionally connected to superior temporal and posterior parietal areas during social-cognitive tasks.^69^^,^^70^ While our fNIRS montage did not allow investigating the contribution of prefrontal regions to third-party social interaction perception, the present findings help to formulate predictions regarding the brain areas and connectivity patterns that might explain the development of social interaction processing.

The study presented here contributes to revealing the fabric of a social brain. We demonstrate neural selectivity for social interactions in occipitotemporal areas of infants as young as 6 and 10 months. These findings show that, beyond an early-developing visual sensitivity to social agents (entities with eyes, faces, bodies, and self-propelled motion), infants by 6 months of age are already sensitive to face-to-face—seemingly interacting—people. The localization of these effects in the infants’ brain corresponds to that found with fMRI, and here with fNIRS, in human adults, pointing to early-developing visual mechanisms for social-interaction perception. Visual areas may encode early, rudimentary representations of social interaction, primarily based on the physical structure of the input (e.g., interpersonal distance and orientation, motion direction). As downstream regions mature and connectivity between posterior and more anterior regions develops, these early representations may support increasingly abstract representations of human social interaction and, more broadly, social relationships.

### Limitations of the study

The neuroimaging technique we utilized, fNIRS, presents many advantages for use in infants, such as safety, tolerance to movement, and smoothness of stimulus presentation.^48^ One important limitation, however, is the lower spatial resolution and specificity when compared with fMRI. Spatial localization of neural effects is based on a probabilistic mapping between fNIRS channels with underlying cortical regions^50^^,^^51^ and the positioning of fNIRS caps according to anatomical landmarks,^49^ which limits the inter-subject correspondence of measured signals. The effect of age (6 vs. 10 months) remains ambiguous. Older infants showed differences in looking time (interacting > non-interacting dyads) that were not observed in younger infants, and neural effects that were visibly—but not statistically—more widespread. Larger samples may help clarify whether developmental changes are present.

## Resource availability

### Lead contact

Requests for further information and resources should be directed to and will be fulfilled by the lead contact, Liuba Papeo (liuba.papeo@isc.cnrs.fr).

### Materials availability

Examples of stimuli generated for and used in our experiments are publicly available on the Open Science Framework, https://doi.org/10.17605/OSF.IO/874RW. Further access to our experimental stimuli will be considered upon request directed to the lead contact.

### Data and code availability

• Pre-processed fNIRS data and infant looking time data that support the findings of this study are publicly available on the Open Science Framework. https://doi.org/10.17605/OSF.IO/874RW.
• The original programming scripts used to pre-process and analyze the data of this study are publicly available on the Open Science Framework. https://doi.org/10.17605/OSF.IO/874RW.
• Any additional information required to re-analyze the data reported in this paper are available from the lead contact upon request.

## Acknowledgments

L.P. was supported by an ANR-DFG Franco-German Grant (FRAL_RELATIONS_268980). J.-R.H. was supported by the 10.13039/501100004431Fondation de France (grant number: 00134704/WB-2022-46104). M.M. was supported by a fellowship of the LabEx CORTEX of the University of Lyon (ANR-11-LABX-0042).

## Author contributions

M.M., conceptualization, methodology, investigation, software, formal analysis, data curation, writing – original draft, writing – review and editing and visualization; E.S., investigation and writing – review and editing; J.-R.H., conceptualization, methodology, writing – original draft; writing – review and editing, funding acquisition, supervision; L.P., conceptualization, methodology, writing – original draft; writing – review and editing, funding acquisition, supervision.

## Declaration of interests

The authors declare no competing interests.

## STAR★Methods

### Key resources table

**Table:** 

REAGENT or RESOURCE	SOURCE	IDENTIFIER
**Deposited data**		

Functional near-infrared spectroscopy data (pre-processed; .pkl)	Open Science Framework	https://doi.org/10.17605/OSF.IO/874RW
Infant looking time data (.csv)	Open Science Framework	https://doi.org/10.17605/OSF.IO/874RW

**Software and algorithms**		

Python – version 3.8.10	Python Software Foundation	https://www.python.org/ - RRID: SCR_008394
MNE Python – version 1.5.0	Gramfort et al.^71^	https://mne.tools/stable/index.html - RRID: SCR_005972
MNE-NIRS – version 0.6.0	Luke et al., 2021^72^	https://mne.tools/mne-nirs/stable/index.html#
R – version 4.3.3	R Core Team	https://www.r-project.org/ - RRID: SCR_001905
RStudio – version 2025.9.0.387	Posit Team	https://posit.co/
Original custom code	Open Science Framework	https://doi.org/10.17605/OSF.IO/874RW

### Experimental models and study participant details

#### Participants

Experiment 1 involved 20 heathy human adults (*M*_age_ = 28.25, *SD*_age_ = 5.41, 11 females), with no report of psychiatric or neurological conditions, and normal or corrected-to-normal vision. They were paid 15 euros for their participation. Two subjects were tested but their data were discarded due to technical problems during the recording session. These participants were replaced to reach a total of 20. This sample size was established based on a previous fNIRS study involving a similar channel-layout and similar stimuli, i.e., point-light displays^73^ (*η*^2^ = 0.1, α = 0.05 and β = 0.8; G∗power 3.1^74^). Experiment 2 involved a total of 41 healthy pre-verbal infants (20 6-month-olds and 21 10-month-olds. 6-month-olds: 11 females, average age = 6 months 15 days; 10-month-olds: 15 females, average age = 10 months 14 days). Infants’ parents gave informed consent and were given a 5€ for reimbursement of travel expenses. A power analysis based on a previous fNIRS study on infants^41^ (*η*^2^ = 0.14; α = 0.05; β = 0.8), showed that a sample size of 15 in each age group would be sufficient to obtain a middle-to-high effect size. To match the number of adult participants, we increased the sample size to 20. Participants or their legal representatives (parents) gave informed consent before taking part in the experiment. Experiments were carried out in accordance with the Declaration of Helsinki (World Medical Association, 2013), and approved by the local ethics committee (CPP Sud-Est II, protocol number: 2023-A00545-40).

Participants’ sex was recorded for general demographic reporting, but this study addresses perceptual mechanisms that are not expected to be influenced by sex, gender and/or sexual orientation. Moreover, we had no *a priori* hypothesis on the effects of these factors on the neural representation of social interaction. Participants’ ancestry, ethnicity, and socioeconomic status were not recorded, as these are considered sensitive data under French law and their collection was therefore not covered by our approval.

### Method details

#### Stimuli

Experiment 1 (adults) involved stimuli used in a previous fMRI study^21^ and consisting of 2-sec video-clips of PLDs depicting two human bodies facing and moving toward or away from each other. Thirteen animations of individual bodies performing various familiar movements were taken from the Communicative Interactions Database (CID^73^). Animations were trimmed so to have all the same duration of 2 sec, and were paired to form 9 interacting dyads, by positioning each pair face-to-face. Non-interacting dyads were created by horizontally flipping the individual bodies in each interacting dyad, yielding the same number of back-to-back dyads. In the two conditions, the two bodies were at matched distance from each other (D_facing_ = 85 pixels ± 82 SD; D_non-facing_ = 92 pixels ± 79 SD; t_(9)_ < 1, n. s.), so that interpersonal orientation and approach (moving toward vs. away from each other) were the only features that differed between interacting and non-interacting dyads (see also supplemental information).

Stimuli for Experiment 2 (study on infants) were similar to those of Experiment 1, except that 9 animations of individual bodies taken from the CID^75^ and were trimmed so that each lasted 5 sec (instead of 2 sec). By pairing individual bodies, eight different facing/interacting and corresponding non-facing/non-interacting dyads were created. The distance between bodies in the dyads was matched between interacting and non-interacting stimuli as done in Experiment 1 (mean_D_facing_ = 199 ± 46, mean_D_non-facing_ = 208 ± 37; t_(7)_ = -1.96, *p* = 0.1) (see also supplemental information for an additional procedure for controlling the distance between the two bodies). Therefore, interpersonal orientation and approach (moving toward vs. away from each another) were the only features that differed between interacting and non-interacting dyads. In addition to body dyads, Experiment 2 also included a set of 2-sec PLDs featuring an individual performing various movement (henceforth, single-body PLDs), taken from the Social Perception and Interaction Database (SoPID^76^).

#### Procedures

In Experiment 1, participants sat on a comfortable chair ∼60 cm from the computer screen for stimulus presentation. This setting was isolated from the rest of the experimental room with black curtains, and monitored throughout the experimental session through a camera connected to the experimenter’s computer, beyond the curtains. After the fNIRS cap montage (∼20 min), participants were instructed to watch the videos on the screen. Stimulus presentation and synchronization with fNIRS recording (Aurora, NIRx Medical Technologies) were controlled by PsychoPy.^77^ Each experimental session consisted of two runs. In the first run, interacting and non-interacting stimuli were shown in random order, in a slow event-related design, interleaved by a jittered inter-trial interval (ITI) between 8 and 12 seconds. A total of 90 stimuli (45 for each condition) were presented in this run, which lasted around 20 minutes. In the second run, participants were presented with a second set of stimuli, for purposes external to the present study. Therefore, data from this second run won’t be discussed further in this article.

In Experiment 2, procedures were identical to Experiment 1 except for the following features. First, infants sat on their parent's laps throughout the fNIRS recording session. Second, stimuli were presented in a slow event-related design, where each trial lasted 10 seconds, consisting of two consecutive presentations of the same 5-sec video-clips for interacting and non-interacting dyads, and five consecutive presentations of the 2-sec video-clips for single-body PLDs. This was done to allow infants enough time to attend to and explore the stimuli. Third, trials were separated by a variable ITI of at least 6 sec, during which two bell sounds of 3 sec each were played to attract the infant’s attention towards the screen. Fourth, stimulus presentation was manually controlled by the experimenter: a trial began when the infant attended to the screen. The entire experimental session lasted around 30 to 45 minutes with a maximum of 48 trials (16 per condition). The experiment was video recorded to allow for offline inspection of infants’ attention to the screen for trial inclusion (see below).

#### fNIRS recording

In Experiment 1, we used an NIRSport2 system (NIRx Medical Technologies; wavelengths: 760nm and 850nm, sampling frequency of 5.1 Hz) with a custom 37-channel array including 16 light sources and 16 light detectors and covering occipital, posterior temporal and parietal cortical regions (Figure 2A). Optodes, and thus channels, arrangement was constructed using the fNIRS Optodes' Location Decider (fOLD) software^50^ and the NIRSite software (NIRx Medical Technologies). fOLD was used to determine the optimal placement of fNIRS optodes on the scalp to maximize anatomical specificity to brain regions of interest. The 10/5 electrode positioning system, with the relative anatomical landmarks, was used as guidance for a detailed coverage of the scalp.^49^ To ensure proper anatomical correspondence between fNIRS channels and underlying cortical regions of interest, fNIRS cap placement during the experiment was based on four reference points, i.e., the nasion, the inion, and the left and right preauricular points.^49^ This step of optode positioning relied on probabilistic correspondences between anatomical landmarks and underlying cortical areas.^50^ Selection of brain regions of interest was informed by the Automated Anatomical Labelling atlas^78^^,^^79^ and the parcellation based on Brodmann Areas.^80^ The so-constructed channel array was then manually inputted in NIRSite and registered on a standard head model (ICBM 152^81^; head circumference = ∼60 cm) to specify the source-detector pairs layout. The average source-detector distance was 34.82 mm (see Tables S1 and S2 for a list of sources, detectors, and channels information). Four different cap sizes were available (54, 56, 58, 60 cm) to best fit the participants’ head.

In Experiment 2, the same fNIRS device was used as in Experiment 1 (NIRSport2 system, NIRx Medical Technologies; wavelengths: 760nm and 850nm, but with a sampling frequency of 5.4 Hz). The following recording properties were specific to infant testing. We used a custom 39-channel array formed by 15 light sources and 15 light detectors and covering occipital, temporal, and parietal cortical regions (Figure 3A). Optodes, and thus channels, arrangement was constructed combining the developmental fNIRS Optodes' Location Decider (devfOLD) software^51^ and the NIRSite software (NIRx Medical Technologies). First, devfOLD was used to automatically determine the optimal placement of fNIRS optodes on the scalp to maximize anatomical specificity to brain regions of interest. The 10/10 electrode positioning system, with the relative anatomical landmarks, was used as guidance for a detailed coverage of the scalp.^49^ Selection of brain regions of interest was informed by the Automated Anatomical Labelling atlas^78^^,^^79^ and the Brainnetome atlas.^82^ The so-constructed channel arrangement was then manually inputted in NIRSite and registered on a standard head model (head circumference = ∼43.4 cm) to specify the source-detector pairs layout. The average source-detector distance was 24.98 mm (see Table S6 and S7 for a list of sources, detectors, and channels information).

### Quantification and statistical analysis

#### Preprocessing of fNIRS data

Data preprocessing was carried out in Python using MNE-NIRS.^71^^,^^72^ Preprocessing involved the following steps. First, individual raw light intensity data were loaded in the MNE-NIRS environment and were converted to optical density (OD) data. Second, OD data were used to calculate the scalp coupling index (sci, range = 0-1) for each channel^83^ and select channels with sci > 0.7; channels that did not satisfy this criterion were interpolated using the ‘nearest-neighbor’ method, the default approach for fNIRS data in MNE (https://mne.tools/stable/generated/mne.io.Raw.html#mne.io.Raw.interpolate_bads) (mean of interpolated channels, 1.55 ± 3.19 channels out of 37). Third, OD data was further cleaned by applying temporal-derivative distribution repair,^84^ an artifact correction procedure based on robust regression, which removes baseline shift and spike artifacts. In addition, motion artifact correction with a custom-made wavelet-based filtering function (wavelet = db5)^85^^,^^86^ was used to remove problematic spike artifacts in the data (see supplemental information, p. 25, for more information on this method; see ref.^87^ for custom-made codes). Fourth, the filtered OD data was then converted into hemoglobin concentration data (HbO/HbR) using the modified Beer-Lambert law, with a partial pathlength factor of 0.1.^72^^,^^88^ HbO/HbR data was band-passed filtered, retaining frequencies between 0.01 Hz (high-pass cutoff) and 0.2 Hz (low-pass cutoff), a range that enclosed our stimulation frequency.^48^ Finally, HbO/HbR signal quality was further improved using a signal enhancement algorithm leveraging the negative correlation between oxygenated and deoxygenated hemoglobin dynamics.^89^

The same preprocessing steps were implemented in Experiment 2, except that we used a partial pathlength factor of 0.5 when converting OD data to hemoglobin concentration data. In infants, an average of 0.34 ± 0.94 channels (out of 39) was interpolated. Additionally, video recordings of the experimental session were parsed offline to select trials in which infants looked at the video for at least half of its duration (5 sec). With this inclusion criterion, an average of 26.88 ± 9.03 trials was retained and entered data preprocessing and analysis.

#### Analyses

Data analyses were carried out with Python (http://www.python.org) and RStudio (Posit team, 2025; https://posit.co/). In both experiments, for each participant, HbO and HbR data were extracted, but only analyses on HbO data is reported in the main text^48^^,^^86^ (see supplemental information for analyses on de-oxyhemoglobin, or HbR, and total hemoglobin concentration, or HbT). HbO data were segmented into epochs from 0 to 14 sec after the stimulus onset, taking into account the average hemodynamic response with a peak around 8 sec (see Figure S7 and ref.^73^). A time window of -2 to 0 seconds relative to the event of interest was used to baseline-correct each epoch. The average response per participant for each channel and for each condition was extracted, and used for two different analyses. In the *channel-wise response analysis*, for each participant, for each channel, we computed the differential HbO concentration [interacting – non-interacting]. The resulting values were entered a no-intercept mixed-effects model with Channel as fixed effect and Participant as random effect^90^ (model specification in supplemental information). This approach yielded model coefficients indicating whether, for each channel, the differential response was significantly different from zero –with values above zero indicating a stronger response to interacting dyads (these model coefficients represent unstandardized effect sizes). For this analysis, model assumptions were tested with the DHARMA package in R^91^ (see supplemental information) and significance level was set at α < 0.05. To complement the channel-wise analysis, one-sample cluster-based permutation tests were run to find ensembles of channels that clustered together in a data-driven fashion. These permutation tests were two-tailed, used a cluster-forming threshold based on a *p*-value of 0.05 for each tail, and were based on channel adjacency matrices of 4-cm and 2.5-cm inter-channel distance in adults and preverbal infants, respectively.

The analyses in Experiment 2 (infants) departed from the general approach presented above in the following ways. The same channel-wise analysis as in Experiment 1 was implemented in Experiment 2, except this was run with Age (6 and 10 months) as a between-subjects factor. Age factor levels were contrast coded as -0.5, for 6-months, and 0.5, for 10-months, so that individual channels’ coefficients would reflect average effects across age groups. For this multi-factorial model, statistical significance of the fixed-effect predictors was assessed with a Type III Wald ANOVA. To explore the representational separability of the social-interaction effect, we proceeded in the following way. We first assessed the absolute response to single-body motion, by testing the channel-wise response to this condition against baseline (zero); we then computed the channel-wise minimum of the t-statistics from the two contrasts (interacting > non-interacting and single > baseline); these minimum t-values were converted into one-sided p-values using the standard normal survival function; by-channel social-interaction and single-body motion effects were then compared to this p-value. This procedure (see also ref.^92^) provided an intuitive measure of overlap in activation strength between the social-interaction and single-body motion effects. Moreover, for the same purpose, i.e., exploring the uniqueness of social interaction perception, we compared the dyad conditions to the single-body motion condition (results reported in supplemental information).

For Experiment 1 (adults), we implemented a Bayesian linear mixed model (with *brms*^93^) replicating the channel-wise analysis in the main analyses. Bayes factors were computed, for each channel parameter, as BF_10_ to assess evidence in favor of the alternative hypothesis (H_1_; contrast [Interacting - Non-interacting] > 0) over the null hypothesis (H_0_), based on prior and posterior samples of a single parameter (using *bayestestR*^94^). We specified an informative prior for the regression coefficients, reflecting theoretical expectations of a positive effect in adults, while allowing for uncertainty and the possibility of negative effects.

In Experiment 2 (infants), for omnibus effects related to the factorial model Channel∗Age in the main analyses, a hierarchical model comparison approach was performed using Bayesian linear mixed models (with *brms*^93^). For infants, we used a weakly informative prior centered on no effect (zero), reflecting no strong prior belief about the direction of the effect. For each channel parameter, Bayes factors were computed as BF_10_ to assess evidence in favor of the alternative hypothesis (H_1_; contrast [Interacting - Non-interacting] ≠ 0) over the null hypothesis (H_0_), and based on prior and posterior samples of a single parameter (using *bayestestR*^94^). The same analysis was also carried out for the contrast [Single-body – baseline].

## Supplemental information

Document S1. Method S1, Figures S1–S9, and Tables S1–S14, additional methodological details and additional supporting data
