# Supplementary material for: Neural selectivity for social interactions in the infant brain
Source: iScience. 2026 Jun 8;29(6):116287. doi: 10.1016/j.isci.2026.116287 (PMC13264107; doi:10.1016/j.isci.2026.116287)
Supplement: Document S1. Method S1, Figures S1–S9, and Tables S1–S14, additional methodological details and additional supporting data [file mmc1.pdf]

iScience, Volume *29*

Supplemental information

Neural selectivity for social interactions in the infant brain

Manuel Mello, Emilie Serraille, Jean-Rémy Hochmann, and Liuba Papeo

Supplemental information

Neural selectivity for social interactions in the infant brain

Manuel Mello, Emilie Serraille, Jean-Rémy Hochmann, Liuba Papeo

Institut des Sciences Cognitives—Marc Jeannerod, UMR5229, Centre National de la Recherche Scientifique (CNRS) and Université Claude Bernard Lyon 1, 67 Bd. Pinel – 69675 Bron (France).

Method S1. Additional methodological details and additional supporting data.

Experiment 1 (Adults)

Below are the fNIRS optode (light sources and detectors; Table S1) and channel (Table S2) specifics for the adult montage, created utilizing the fNIRS Optodes' Location Decider (fOLD) software (Zimeo Morais et al., 2018) and the NIRSite software (NIRx Medical Technologies).

Table S1: Optodes characteristics in adult fNIRS montage. S = light source, D = light detector.

**Sources**			**Detectors**		
**Optode**	**Landmark**	**Location (x, y, z)**	**Optode**	**Landmark**	**Location (x, y, z)**
S01	POO2	14, -114, 32	D01	PPO2h	13, -96, 68
S02	POO10h	45, -107, -17	D02	PPO6h	57, -93, 36
S03	TPP10h	78, -59, -30	D03	PPO10h	66, -86, -24
S04	CPP6h	73, -63, 47	D04	TPP8h	79, -61, 9
S05	CCP4h	57, -28, 82	D05	CPP4h	52, -66, 77
S06	TTP8h	86, -29, 7	D06	CPP6h	81, -29, 48
S07	FTT10h	82, -1, -37	D07	FTT8h	82, 2, 3
S08	OI1h	-15, -120, -9	D08	FFT10h	77, 29, -39
S09	PPO1h	-14, -96, 68	D09	OI2h	15, -120, -9
S10	CPP3h	-52, -66, 77	D10	POO1	-14, -114, 31
S11	PPO5h	-57, -93, 36	D11	POO9h	-45, -108, -18
S12	PPO9h	-65, -86, -25	D12	CPP5h	-73, -65, 46
S13	TPP7h	-79, -62, 9	D13	TPP9h	-78, -60, -30
S14	CCP5h	-81, -30, 47	D14	TTP7h	-86, -31, 6
S15	FTT7h	-83, 1, 2	D15	CCP3h	-58, -29, 81
S16	FFT9h	-78, 28, -40	D16	FTT9h	-82, -2, -38

Table S2: Channel characteristics in adult fNIRS montage. *Abbreviations.* CUN: cuneus; I/M/SOG: inferior/middle/superior occipital gyrus; CAL: calcarine fissure and surrounding cortex; CERCRU1 = Crus I of cerebellar hemisphere; LING = lingual gyrus; I/M/STG = inferior/middle/superior temporal gyrus; ANG = angular gyrus; I/SPG = inferior/superior parietal gyrus; SMG = supramarginal gyrus; TPOmid = temporal pole: middle temporal gyrus.

**S-D pair**	**Landmarks**	**AAL2**	**Brodmann**	**MNI coordinates (x, y, z)**
S01-D01	POO2-PPO2h	CUN (R) (47%)<br>SOG (R) (28%)	V2 (BA18) (R) (47%)<br>V3 (BA19) (R) (30%)	11, -90, 27

S01-D10	POO2-POO1	SOG (L) (32%)<br>CUN (L) (26%)	V1 (BA17) (L) (73%)	-3, -99, 12
S01-D09	POO2-OI2h	CAL (R) (36%)	V1 (BA17) (R) (63%)<br>V2 (BA18) (R) (28%)	13, -98, 0
S02-D03	POO10h-PPO10h	CERCRU1 (R) (49%)<br>IOG (R) (21%)	V3 (BA19) (R) (48%)	41, -79, -23
S02-D09	POO10h-OI2h	LING (R) (35%)<br>IOG (R) (23%)	V2 (BA18) (R) (65%)	25, -92, -17
S03-D03	TPP10h-PPO10h	CERCRU1 (R) (49%)<br>ITG (R) (37%)	FFG (BA37) (R) (65%)	53, -61, -26
S03-D04	TPP10h-TPP8h	ITG (R) (53%)<br>MTG (R) (45%)	FFG (BA37) (R) (64%)	62, -52, -8
S04-D02	CPP6h-PPO6h	ANG (R) (47%)<br>MOG (R) (42%)	ANG (BA39) (R) (47%)	48, -71, 31
S04-D04	CPP6h-TPP8h	MTG (R) (45%)<br>ANG (R) (36%)	ANG (BA39) (R) (38%)<br>FFG (BA37) (R) (22%)	58, -58, 22
S04-D05	CPP6h-CPP4h	ANG (R) (63%)<br>IPG (R) (26%)	ANG (R) (63%)	47, -62, 47
S04-D06	CPP6h-CCP6h	SMG (R) (42%)<br>IPG (R) (32%)<br>ANG (R) (24%)	SMG (BA40) (R) (66%)<br>ANG (BA39) (R) (22%)	58, -47, 38
S05-D05	CCP4h-CPP4h	SPG (R) (50%)	SMG (BA40) (R) (39%)	39, -50, 60
S05-D06	CCP4h-CCP6h	SPG (R) (39%)<br>SMG (R) (28%)	SMG (BA40) (R) (52%)	53, -36, 51
S06-D04	TTP8h-TPP8h	MTG (R) (76%)<br>STG (R) (21%)	STG (BA22) (R) (50%)<br>MTG (BA21) (R) (39%)	65, -44, 6
S06-D06	TTP8h-CCP6h	SMG (R) (49%)<br>STG (R) (43%)	STG (BA22) (R) (35%)	65, -33, 22
S06-D07	TTP8h-FTT8h	STG (R) (73%)	STG (BA22) (R) (60%)<br>MTG (BA21) (R) (30%)	67, -20, 5
S07-D07	FTT10h-FTT8h	MTG (R) (52%)<br>STG (R) (35%)	MTG (BA21) (R) (75%)	65, -7, -12
S07-D08	FTT10h-FFT10h	MTG (R) (48%)<br>TPOmid (R) (30%)	MTG (BA21) (R) (75%)	59. 4, -25
S08-D10	OI1h-POO1	MOG (L) (41%)<br>CAL (L) (28%)	V1 (BA17) (L) (73%)	-14, -101, 0
S08-D11	OI1h-POO9h	LING (L) (41%)<br>IOG (L) (22%)	V2 (BA18) (L) (67%)	-24, -93, -26
S08-D09	OI1h-OI2h	CAL (L) (37%)	V2 (BA18) (L) (57%)<br>V3 (BA19) (L) (27%)	-3, -95, -14
S09-D01	PPO1h-PPO2h		V3 (BA19) (L) (50%)	-1, -82, 44
S09-D10	PPO1h-POO1	SOG (L) (52%)<br>CUN (L) (37%)	V2 (BA18) (L) (43%)<br>V3 (BA19) (L) (29%)	-11, -92, 28
S10-D12	CPP3h-CPP5h	ANG (L) (50%)<br>IPG (L) (40%)	ANG (BA39) (L) (63%)	-46, -62, 46
S10-D15	CPP3h-CCP3h	SPG (L) (35%)<br>IPG (L) (33%)	SMG (BA40) (L) (34%)	-39, -49, 60

S11-D12	PPO5h-CPP5h	ANG (L) (58%)<br>MOG (L) (31%)	ANG (BA39) (L) (81%)	-47, -71, 30
S12-D11	PPO9h-POO9h	CERCRU1 (L) (50%)	V3 (BA19) (L) (49%)	-41, -77, -21
S12-D13	PPO9h-TPP9h	ITG (L) (51%)<br>CERCRU1 (L) (34%)	FFG (BA37) (L) (55%)	-56, -58, -23
S13-D12	TPP7h-CPP5h	MTG (L) (48%)<br>ANG (L) (29%)	ANG (BA39) (L) (38%)<br>FFG (BA37) (L) (24%)	-57, -58, 21
S13-D13	TPP7h-TPP9h	MTG (L) (60%)<br>ITG (L) (35%)	FFG (BA37) (L) (63%)<br>ITG (BA20) (L) (21%)	-64, -51, -9
S13-D14	TPP7h-TTP7h	MTG (L) (83%)	MTG (BA21) (L) (42%)<br>STG (BA22) (L) (37%)	-65, -44, 4
S14-D12	CCP5h-CPP5h	IPG (L) (52%)<br>SMG (L) (34%)	SMG (BA40) (L) (64%)<br>ANG (BA39) (L) (22%)	-57, -47, 37
S14-D14	CCP5h-TTP7h	STG (L) (28%)	SMG (BA40) (L) (46%)<br>STG (BA22) (L) (31%)	-64, -33, 22
S14-D15	CCP5h-CCP3h	IPG (L) (52%)	SMG (BA40) (L) (44%)	-53, -34, 51
S15-D14	FTT7h-TTP7h	MTG (L) (54%)<br>STG (L) (32%)	STG (BA22) (L) (47%)<br>MTG (BA21) (L) (39%)	-66, -20, 4
S15-D16	FTT7h-FTT9h	MTG (L) (67%)<br>STG (L) (21%)	MTG (BA21) (L) (69%)	-62, -7, -12
S16-D16	FFT9h-FTT9h	MTG (L) (64%)	MTG (BA21) (L) (72%)	-57, 2, -25

Table S3 summarizes the characteristics (10-5 system landmarks, correspondence with underlying cortical area with specificity, and location) of channels that grouped together in our cluster-based permutation analysis in Experiment 1 in adults.

Table S3. Channels clustering together in our cluster-based permutation analysis of Experiment 1 (adults).

**Channel**	**AAL**	**MNI coordinates (x, y, z)**	**Cluster**
TTP8h-TPP8h	MTG (R) (76%)<br>STG (R) (21%)	65, -44, 6	Cluster #1, right hemisphere
TTP8h-FTT8h	STG (R) (73%)	67, -20, 5	
TTP8h-CCP6h	SMG (R) (49%)<br>STG (R) (43%)	65, -33, 22	
TPP10h-TPP8h	ITG (R) (53%)<br>MTG (R) (45%)	62, -52, -8	
CPP6h-CCP6h	SMG (R) (42%)<br>IPG (R) (32%)<br>ANG (R) (24%)	58, -47, 38	
CCP4h-CPP4h	SPG (R) (50%)	39, -50, 60	

CCP4h-CCP6h	SPG (R) (39%)<br>SMG (R) (28%)	53, -36, 51	
CPP6h-PPO6h	ANG (R) (47%)<br>MOG (R) (42%)	48, -71, 31	
CPP6h-CPP4h	ANG (R) (63%)<br>IPG (R) (26%)	47, -62, 47	
CPP6h-TPP8h	MTG (R) (45%)<br>ANG (R) (36%)	58, -58, 22	
PPO5h-CPP5h	ANG (L) (58%)<br>MOG (L) (31%)	-47, -71, 30	Cluster #2, left hemisphere
TPP7h-CPP5h	MTG (L) (48%)<br>ANG (L) (29%)	-57, -58, 21	
TPP7h-TPP9h	MTG (L) (60%)<br>ITG (L) (35%)	-64, -51, -9	
TPP7h-TTP7h	MTG (L) (83%)	-65, -44, 4	
CCP5h-CPP5h	IPG (L) (52%)<br>SMG (L) (34%)	-57, -47, 37	
CPP3h-CPP5h	ANG (L) (50%)<br>IPG (L) (40%)	-46, -62, 46	
CCP5h-TTP7h	SMG (L) (46%)<br>STG (L) (31%)	-64, -33, 22	

Note. **“**Channel” labels denote landmarks of the 10-5 electrode positioning system (Jurcak et al., 2007). AAL labels reflect probabilistic mapping of underlying cortical areas (specificity in parenthesis) as calculated in Zimeo Morais et al. (2018). MOG = inferior/middle occipital gyrus; I/M/STG = inferior/middle/superior temporal gyrus; ANG = angular gyrus; I/SPG = inferior/superior parietal gyrus; SMG = supramarginal gyrus.

Experiment 1. Additional analyses

Channel-wise response analysis. Below, we report the model specification formula (using *lmer*; Bates et al., 2015) for our channel-wise response analysis as well as our procedure for assessing model assumptions using DHARMA (Hartig, 2024).

Model specification:

```
channelwise_model = lmer(Difference ~ -1 + ch_name + (1|ID), data=data,
        REML=T,
        control=lmerControl(check.conv.singular = .makeCC(action = "ignore",  tol = 1e-4)))
```

Model assumptions:

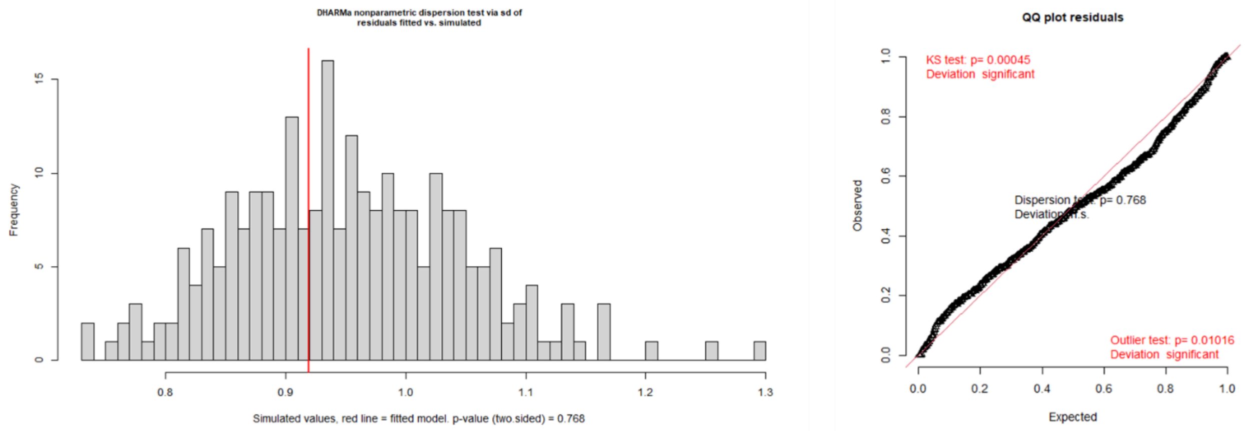

Figure S1: Model assumptions for the channel-wise response analysis. Residuals dispersion was met (p = 0.77), while model residuals showed a slight deviation from normality (p < 0.001) and the presence of outliers (p = 0.01), possibly due to high number of data points. In fact, note that *"the p-value alone is not a good indicator of the extent to which your residuals deviate from assumptions. Specifically, if you have a lot of data points, residual diagnostics will nearly inevitably become significant, because having a perfectly fitting model is very unlikely. That, however, doesn't necessarily mean that you need to change your model"* (Hartig, 2024).

Because normality of residuals (p < 0.001) and presence of outliers (p = 0.01) were not met for this model, we re-run the model using robust mixed modeling (Koller, 2016).

Model specification:

robust_channelwise_model <- rlmer(Difference ~ -1 + ch_name + (1 | ID), data = data)

Results were congruent with those reported in the main text, in terms of significant channels for the [interacting – non-interacting] contrast (Table S4).

Table S4: Channels with significant effect in the channel-wise response analysis – social interaction contrast in adults, using robust mixed modeling.

Channel	Statistics	AAL2	MNI coordinates (x, y, z)
**CPP6h-PPO6h**	***est.*** **= 1.12 ± 0.37,** $t_{(182.7)}$ **= 3.04,** p **= 0.002**	**ANG (R) (47%)**<br>**MOG (R) (42%)**	**48, -71, 31**
**CPP6h-TPP8h**	***est.*** **= 0.88 ± 0.37,** $t_{(182.7)}$ **= 2.39,** p **= 0.01**	**MTG (R) (45%)**<br>**ANG (R) (36%)**	**58, -58, 22**
**CPP6h-CPP4h**	***est.*** **= 1.06 ± 0.37,** $t_{(182.7)}$ **= 2.86,** p **= 0.004**	**ANG (R) (63%)**<br>**IPG (R) (26%)**	**47, -62, 47**
**CPP6h-CCP6h**	***est.*** **= 0.8 ± 0.37,** $t_{(182.7)}$ **= 2.16,** p **= 0.03**	**SMG (R) (42%)**<br>**IPG (R) (32%)**<br>**ANG (R) (24%)**	**58, -47, 38**
**CCP4h-CPP4h**	***est.*** **= 1 ± 0.37,** $t_{(182.7)}$ **= 2.71,** p **= 0.007**	**SPG (R) (50%)**	**39, -50, 60**
**CCP4h-CCP6h**	***est.*** **= 1.2 ± 0.37,** $t_{(182.7)}$ **= 3.26,** p **= 0.001**	**SPG (R) (39%)**<br>**SMG (R) (28%)**	**53, -36, 51**

**TTP8h-TPP8h**	***est.* = 0.9 ± 0.37, $t_{(182.7)}$ = 2.43, *p* = 0.01**	**MTG (R) (76%)<br>STG (R) (21%)**	**65, -44, 6**
**TTP8h-CCP6h**	***est.* = 0.79 ± 0.37, $t_{(182.7)}$ = 2.15, *p* = 0.03**	**SMG (R) (49%)<br>STG (R) (43%)**	**65, -33, 22**
**CPP3h-CPP5h**	***est.* = 0.76 ± 0.37, $t_{(182.7)}$ = 2.04, *p* = 0.04**	**ANG (L) (50%)<br>IPG (L) (40%)**	**-46, -62, 46**
**PPO5h-CPP5h**	***est.* = 1.19 ± 0.37, $t_{(182.7)}$ = 3.21, *p* = 0.001**	**ANG (L) (58%)<br>MOG (L) (31%)**	**-47, -71, 30**
**TPP7h-CPP5h**	***est.* = 1.25 ± 0.37, $t_{(182.7)}$ = 3.72, *p* < 0.001**	**MTG (L) (48%)<br>ANG (L) (29%)**	**-57, -58, 21**
**TPP7h-TPP9h**	***est.* = 0.99 ± 0.37, $t_{(182.7)}$ = 2.69, *p* = 0.007**	**MTG (L) (60%)<br>ITG (L) (35%)**	**-64, -51, -9**
**TPP7h-TTP7h**	***est.* = 0.78 ± 0.37, $t_{(182.7)}$ = 2.11, *p* = 0.03**	**MTG (L) (83%)**	**-65, -44, 4**
**CCP5h-CPP5h**	***est.* = 1.31 ± 0.37, $t_{(182.7)}$ = 3.54, *p* < 0.001**	**IPG (L) (52%)<br>SMG (L) (34%)**	**-57, -47, 37**
**CCP5h-CCP3h**	***est.* = 1.11 ± 0.37, $t_{(182.7)}$ = 3.01, *p* = 0.002**	**IPG (L) (52%)**	**-53, -34, 51**
TPP10h-TPP8h	*est.* = 0.8 ± 0.37, $t_{(182.7)}$ = 2.17, *p* = 0.03	ITG (R) (53%)<br>MTG (R) (45%)	62, -52, -8
OI1h-POO1	*est.* = 0.78 ± 0.37, $t_{(182.7)}$ = 2.01, *p* = 0.03	MOG (L) (41%)<br>CAL (L) (28%)	-14, -101, 0
CPP3h-CCP3h	*est.* = 0.91 ± 0.37, $t_{(182.7)}$ = 2.47, *p* = 0.01	SPG (L) (35%)<br>IPG (L) (33%)<br>PoCG (L) (29%)	-39, -49, 60

Note. Highlighted in bold are the channel that were significantly activated in both analyses (non-robust and robust mixed modeling). CERCRU1 = Crus I of cerebellar hemisphere; MOG = middle occipital gyrus; I/M/STG = inferior/middle/superior temporal gyrus; ANG = angular gyrus; I/SPG = inferior/superior parietal gyrus; SMG = supramarginal gyrus; CAL = calcarine fissure and surrounding cortex; PoCG = postcentral gyrus.

Analyses on deoxyhemoglobin (HbR) and total hemoglobin concentration (HbT)

In addition to the analysis on the HbO concentration (main text), here we report the channel-wise and ROI-based analyses carried out on HbR and HbT (defined as HbO + HbR).

Channel-wise analysis. Modeling HbR for the [interacting > non-interacting] contrast revealed an effect in three channels, whereby differential HbR concentration was significantly lower for interacting dyads (Table 5). Similar to HbO results, modeling HbT for the [interacting > non-interacting] contrast revealed an effect in seventeen channels, whereby HbT concentration was significantly higher for interaction perception (Table S5).

Table S5: Channels with significant effect in the channel-wise response analysis on HbR and HbT concentration – social interaction contrast in adults.

Channel	Statistics	AAL2	MNI coordinates (x, y, z)
**HbR**			
POO10h-PPO10h	*est.* = -0.87 ± 0.2, $t_{(497.78)}$ = -4.83, p < 0.001	CERCRU1 (R) (49%)<br>IOG (R) (21%)	41, -79, -23
CCP4h-CCP6h	*est.* = -0.44 ± 0.2, $t_{(497.78)}$ = -2.22, p = 0.02	IPG (R) (39%)<br>SMG (R) (28%)	53, -36, 51
CCP5h-CPP5h	*est.* = -0.41 ± 0.2, $t_{(497.78)}$ = -2.05, p = 0.05	IPG (L) (52%)<br>SMG (L) (34%)	-57, -47, 37
**HbT**			
TPP10h-TPP8h	*est.* = 0.59 ± 0.27, $t_{(134.7)}$ = 2.19, p = 0.03	ITG (R) (53%)<br>MTG (R) (45%)	58, -47, 38
CPP6h-PPO6h	*est.* = 0.97 ± 0.27, $t_{(134.7)}$ = 3.19, p = 0.002	ANG (R) (47%)<br>MOG (R) (42%)	48, -71, 31
CPP6h-TPP8h	*est.* = 0.75 ± 0.27, $t_{(134.7)}$ = 2.74, p = 0.007	MTG (R) (50%)<br>ANG (R) (36%)	58, -58, 22
CPP6h-CPP4h	*est.* = 0.83 ± 0.27, $t_{(134.7)}$ = 3.05, p = 0.003	ANG (R) (63%)<br>IPG (R) (26%)	47, -62, 47
CPP6h-CCP6h	*est.* = 0.65 ± 0.27, $t_{(134.7)}$ = 2.38, p = 0.01	SMG (R) (42%)<br>IPG (R) (32%)<br>ANG (32%)	58, -47, 38
CCP4h-CPP4h	*est.* = 0.74 ± 0.27, $t_{(134.7)}$ = 2.74, p = 0.007	SPG (R) (50%)<br>PoCG (R) (23%)	39, -50, 60
CCP4h-CCP6h	*est.* = 0.87 ± 0.27, $t_{(134.7)}$ = 3.21, p = 0.002	IPG (R) (39%)<br>SMG (R) (28%)	53, -36, 51
TTP8h-TPP8h	*est.* = 0.74 ± 0.27, $t_{(134.7)}$ = 2.74, p = 0.007	MTG (R) (76%)<br>STG (R) (21%)	65, -44, 6
TTP8h-CCP6h	*est.* = 0.58 ± 0.27, $t_{(134.7)}$ = 2.15, p = 0.03	SMG (R) (49%)<br>STG (R) (43%)	65, -33, 22
OI1h-POO1	*est.* = 0.56 ± 0.27, $t_{(134.7)}$ = 2.08, p = 0.03	MOG (L) (41%)<br>CAL (L) (28%)	-14, -101, 0
CPP3h-CPP5h	*est.* = 0.58 ± 0.27, $t_{(134.7)}$ = 2.13, p = 0.03	ANG (L) (50%)<br>IPG (L) (40%)	-46, -62, 46
PPO5h-CPP5h	*est.* = 0.9 ± 0.27, $t_{(134.7)}$ = 3.3, p = 0.001	ANG (L) (58%)<br>MOG (L) (31%)	-47, -71, 30
TPP7h-CPP5h	*est.* = 0.95 ± 0.27, $t_{(134.7)}$ = 3.49, p = 0.001	MTG (L) (48%)<br>ANG (L) (29%)	-57, -58, 21
TPP7h-TPP9h	*est.* = 0.76 ± 0.27, $t_{(134.7)}$ = 2.78, p = 0.006	MTG (L) (60%)<br>ITG (L) (35%)	-64, -51, -9
TPP7h-TTP7h	*est.* = 0.6 ± 0.27, $t_{(134.7)}$ = 2.21, p = 0.02	MTG (L) (83%)	-65, -44, 4

CCP5h-CPP5h	*est.* = 0.86 ± 0.27, $t_{(134.7)}$ = 3.16, *p* = 0.002	IPG (L) (52%)<br>SMG (L) (34%)	-57, -47, 37
CCP5h-CCP3h	*est.* = 0.56 ± 0.27, $t_{(134.7)}$ = 2.05, *p* = 0.04	IPG (L) (52%)<br>PoCG (L) (35%)	-53, -34, 51

Note. CERCRU1 = Crus I of cerebellar hemisphere; MOG = middle occipital gyrus; I/M/STG = inferior/middle/superior temporal gyrus; ANG = angular gyrus; I/SPG = inferior/superior parietal gyrus; SMG = supramarginal gyrus; CAL = calcarine fissure and surrounding cortex; PoCG = postcentral gyrus.

Experiment 2 (Infants)

Below are the fNIRS optode (light sources and detectors; Table S6) and channel (Table S7) specifics for the infant montage, created utilizing the developmental fNIRS Optodes' Location Decider (devfOLD) software (Fu and Richards, 2021) and the NIRSite software (NIRx Medical Technologies).

Table S6: Optodes characteristics in infant fNIRS montage.

Sources			Detectors		
**Optode**	**Landmark**	**Location (x, y, z)**	**Optode**	**Landmark**	**Location (x, y, z)**
S01	POz	0, -59, 33	D01	Oz	0, -68, 9
S02	O2	22, -66, 3	D02	PO4	29, -57, 25
S03	PO10	38, -50, -30	D03	PO8	41, -54, -3
S04	P4	42, -39, 36	D04	P6	52, -37, 15
S05	P8	52, -35, -8	D05	CP4	49, -15, 41
S06	CP6	59, -14, 16	D06	TP8	59, -13, -12
S07	TP10	56, -10, -40	D07	C6	59, 10, 13
S08	T8	60, 10, -15	D08	FT8	56, 30, -16
S09	O1	-22, -66, 3	D09	PO3	-29, -58, 24
S10	PO9	-37, -51, -30	D10	PO7	-41, -54, -3
S11	P7	-53, -35, -8	D11	P5	-52, -37, 15
S12	P3	-42, -39, 36	D12	TP7	-59, -13, -12
S13	CP5	-59, -14, 16	D13	CP3	-49, -15, 41
S14	TP9	-55, -11, -39	D14	C5	-59, 10, 13
S15	T7	-60, 10, -15	D15	FT7	-55, 30, -16

Note. S = light source, D = light detector.

Table S7: Channel characteristics in infant fNIRS montage.

S-D pair	Landmarks	AAL2	Brainnetome	Coordinates (x, y, z)
S01-D01	POz-Oz	CUN (R) (41%)<br>CAL (R) (27%)<br>SOG (R) (23%)	LOG (R) (49%)	-3, -88, 22
S01-D02	POz-PO4	SOG (R) (58%)<br>CUN (R) (25%)	LOG (R) (69%)	11, -84, 33
S01-D09	POz-PO3	SOG (L) (63%)<br>CUN (L) (23%)	LOG (L) (64%)	-16, -82, 32
S02-D01	O2-Oz	MOG (R) (51%)<br>CAL (R) (26%)	LOG (R) (87%)	10, -91, 9

S02-D02	O2-PO4	MOG (R) (88%)	LOG (R) (90%)	22, -86, 19
S02-D03	O2-PO8	MOG (R) (88%)	LOG (R) (95%)	30, -84, 7
S03-D03	PO10-PO8	CERCRU1 (R) (39%)<br>MOG (R) (23%)<br>IOG (R) (21%)	LOG (R) (76%)	38, -77, -6
S04-D02	P4-PO4	MOG (R) (61%)<br>ANG (R) (26%)	IPG (BA39) (R) (86%)	32, -75, 36
S04-D04	P4-P6	ANG (R) (78%)	IPG (BA39) (R) (75%)	45, -65, 32
S04-D05	P4-CP4	IPG (R) (63%)<br>ANG (R) (32%)	IPG (BA40) (R) (52%)<br>IPG (BA39) (R) (25%)	44, -54, 47
S05-D03	P8-PO8	IOG (R) (36%)<br>MOG (R) (29%)	LOG/V5 (R) (32%)<br>ITG (BA37) (R) (23%)	46, -69, 2
S05-D04	P8-P6	MTG (R) (68%)	MTG (BA37) (R) (49%)<br>ITG (BA37) (R) (24%)	51, -61, 11
S05-D06	P8-TP8	MTG (R) (55%)<br>ITG (R) (45%)	MTG (BA21) (R) (34%)<br>ITG (BA37) (R) (25%)	56, -49, -4
S06-D04	CP6-P6	MTG (R) (37%)<br>STG (R) (31%)	STG (BA22) (R) (32%)<br>MTG (BA37) (R) (24%)	55, -52, 22
S06-D05	CP6-CP4	SMG (R) (42%)<br>IPG (R) (42%)	IPG (BA40) (R) (62%)	56, -42, 36
S06-D06	CP6-TP8	MTG (R) (78%)	MTG (BA21) (R) (41%)<br>STG (BA22) (R) (28%)	60, -39, 7
S06-D07	CP6-C6	PoCG (R) (39%)<br>STG (R) (32%)	STG (BA22) (R) (31%)<br>PoCG (R) (22%)	61, -28, 20
S07-D06	TP10-TP8	ITG (R) (56%)<br>MTG (R) (36%)	MTG (BA21) (R) (38%)<br>ITG (BA20) (R) (34%)	57. -41, -18
S08-D06	TP8-TP8	MTG (R) (71%)<br>ITG (R) (29%)	MTG (BA21) (R) (62%)	61, -29, -11
S08-D07	T8-C6	MTG (R) (48%)<br>STG (R) (21%)	aSTS (R) (21.1%)	62, -15, 3
S08-D08	T8-FT8	MTG (R) (70%)	MTG (BA21) (R) (52%)	61, -7, -12
S09-D01	O1-Oz	CAL (L) (31%)	LOG (L) (88.2%)	-13, -90, 8
S09-D10	O1-PO7	MOG (L) (49%)<br>IOG (L) (44%)	LOG (L) (77.3%)	-33, -81, 6
S09-D09	O1-PO3	MOG (L) (56%)<br>SOG (L) (30%)	LOG (L) (96%)	-26, -84, 17
S10-D10	PO9-PO7	IOG (L) (50%)<br>CERCRU1 (L) (31%)	IOG (L) (72%)	-40, -74, -8
S11-D10	P7-PO7	ITG (L) (38%)<br>IOG (L) (33%)	LOG/V5/MT (L) (47%)	-48, 67, 0
S11-D11	P7-P5	MTG (L) (72%)<br>ITG (L) (22%)	MTG (BA37) (L) (54%)	-53, -59, 9
S11-D12	P7-TP7	ITG (L) (68%)<br>MTG (L) (31%)	MTG (BA21) (L) (34%)<br>ITG (BA20) (L) (24%)<br>MTG (BA37) (L) (23%)	-56, -46, -6

S12-D11	P3-P5	ANG (L) (61%)<br>MTG (L) (20%)	IPG (BA39) (L) (80%)	-48, -63, 30
S12-D13	P3-CP3	IPG (L) (51%)<br>ANG (L) (39%)	IPG (BA40) (L) (44%)<br>ITG (BA39) (L) (30%)	-49, -52, 44
S12-D09	P3-PO3	MOG (L) (51%)<br>ANG (L) (32%)	IPG (BA39) (L) (78%)	-36, -73, 34
S13-D11	CP5-P5	MTG (L) (42%)<br>STG (L) (34%)	IPG (BA39) (L) (34%)<br>IPG (BA40) (L) (30%)	-57, -49, 20
S13-D12	CP5-TP7	MTG (L) (57%)<br>STG (L) (31%)	MTG (BA21) (L) (42%)<br>STG (BA22) (L) (24%)	-60, -36, 5
S13-D13	CP5-CP3	SMG (L) (58%)<br>IPG (L) (22%)	IPG (BA40) (L) (75%)	-58, -39, 33
S13-D14	CP5-C5	STG (L) (52%)<br>SMG (L) (27%)	STG (BA22) (L) (39%)	-62, -26, 17
S14-D12	TP9-TP7	ITG (L) (65%)	ITG (BA20) (L) (51%)<br>MTG (BA21) (L) (37%)	-56, -38, -20
S15-D12	T7-TP7	MTG (L) (64%)<br>ITG (L) (33%)	MTG (BA21) (L) (70%)	-60, -26, -13
S15-D14	T7-C5	STG (L) (49%)<br>MTG (L) (28%)	STG (BA21) (L) (72%)<br>aSTS (L) (23%)	-61, -14, 0
S15-D15	T7-FT7	MTG (L) (53%)	MTG (BA21) (L) (30%)<br>aSTS (L) (29%)	-59, -5, -14

Note. Abbreviations. CUN: cuneus; I/M/SOG: inferior/middle/superior occipital gyrus; CAL: calcarine fissure and surrounding cortex; CERCRU1 = Crus I of cerebellar hemisphere; LING = lingual gyrus; I/M/STG = inferior/middle/superior temporal gyrus; ANG = angular gyrus; I/SPG = inferior/superior parietal gyrus; SMG = supramarginal gyrus; TPOmid = temporal pole: middle temporal gyrus; PoCG = postcentral gyrus.

Table S8 summarizes the characteristics (10-10 system landmarks, correspondence with underlying cortical area with specificity, and location) of channels that grouped together in our cluster-based permutation analysis in Experiment 2 in infants.

Table S8. Channels clustering together in our cluster-based permutation analysis of Experiment 2 (infants).

Channel	AAL	Coordinates (x, y, z)	Cluster
P4-PO4	MOG (R) (61%)<br>ANG (R) (26%)	32, -75, 36	Cluster #1, right hemisphere
P4-P6	ANG (R) (78%)	45, -65, 32	
CP6-CP4	SMG (R) (42%)<br>IPG (R) (42%)	56, -42, 36	
P4-CP4	IPG (R) (63%)<br>ANG (R) (32%)	44, -54, 47	
CP6-P6	MTG (R) (37%)<br>STG (R) (31%)	55, -52, 22	

CP5-CP3	SMG (L) (58%)<br>IPG (L) (22%)	-58, -39, 33	Cluster #2, left hemisphere
CP5-P5	MTG (L) (42%)<br>STG (L) (34%)	-57, -49, 20	
P7-P5	MTG (L) (72%)<br>ITG (L) (22%)	-53, -59, 9	
CP5-TP7	MTG (L) (57%)<br>STG (L) (31%)	-60, -36, 5	
P7-TP7	ITG (L) (68%)<br>MTG (L) (31%)	-56, -46, -6	
P3-CP3	IPG (L) (51%)<br>ANG (L) (39%)	-49, -52, 44	

Note. "Channel" labels denote landmarks of the 10-10 electrode positioning system (Jurcak et al., 2007). AAL labels reflect probabilistic mapping of underlying cortical areas (specificity in parenthesis) as calculated in Fu and Richards (2021). MOG = middle occipital gyrus; I/M/STG = inferior/middle/superior temporal gyrus; SMG = supramarginal gyrus; ANG = angular gyrus; IPG = inferior parietal gyrus; AAL labels represent probabilistic mapping of underlying cortical areas (specificity in parenthesis).

Additional analyses – infants

In the following series of analyses on infant fNIRS data, we explored neural sensitivity to social interactions and to single-body motion in 6- and 10-month-olds separately

Six-month-olds. The fNIRS map resulting from the contrast interacting > non-interacting stimuli was subjected to a no-intercept mixed linear model with Channel as fixed effect and Participant as random factor. The results showed an effect of social interaction (i.e., above zero difference for interacting – non-interacting) in a channel encompassing the middle occipital and angular gyrus, $t_{(226.95)} = 2.05$, $p = 0.04$, est. = 1.96 ± 0.96 (Fig. S3A and Table S9). A one-sample cluster-based permutation on the selectivity values [interacting – non-interacting] determined channels that clustered together in a data-driven way. This analysis revealed no significant clusters in 6-month-olds (Fig. S3B).

The analysis modelling channel-wise activation for single-body motion perception against baseline revealed an effect in ten channels (all ts > 2.03, all ps < 0.05; Fig. S3C) covering the middle occipital and temporal cortex bilaterally, with no overlap with the effect of the contrast interacting > non-interacting. Additionally, we directly compared the effects of dyad-perception against perception of single-body, which confirmed the spatial segregation of the two effects (Fig. S3D). Modeling HbO concentration for the [interacting > single-body] contrast in 6-month-olds revealed an effect in two channels. P3-CP3 (left inferior parietal/angular cortex) showed higher activity for interacting dyads (est. = 1.64 ± 0.76, $t_{(228.09)} = 2.15$, $p = 0.03$), while O2-PO4 (right middle occipital cortex) showed higher activity for single-bodies (est. = -1.72 ± 0.76, $t_{(228.09)} = -2.25$, $p = 0.02$). Modeling HbO concentration for the [non-interacting > single-body] contrast in 6-month-olds revealed no effects. Modeling HbO concentration for the [dyads > single-body] contrast in 6-month-olds revealed an effect in two channels, for which activity was higher for single-bodies: O2-PO4

(right middle occipital cortex; est. = -1.62 ± 0.66, $t_{(309.31)}$ = -2.46, p = 0.01) and O2-PO8 (right middle occipital cortex; est. = -1.35 ± 0.66, $t_{(309.31)}$ = -2.06, p = 0.04). In sum, these analyses in 6-month-old infants revealed selectivity to social interactions (when compared to non-interacting dyads and single-body motion) in the left middle occipital/inferior parietal cortex.

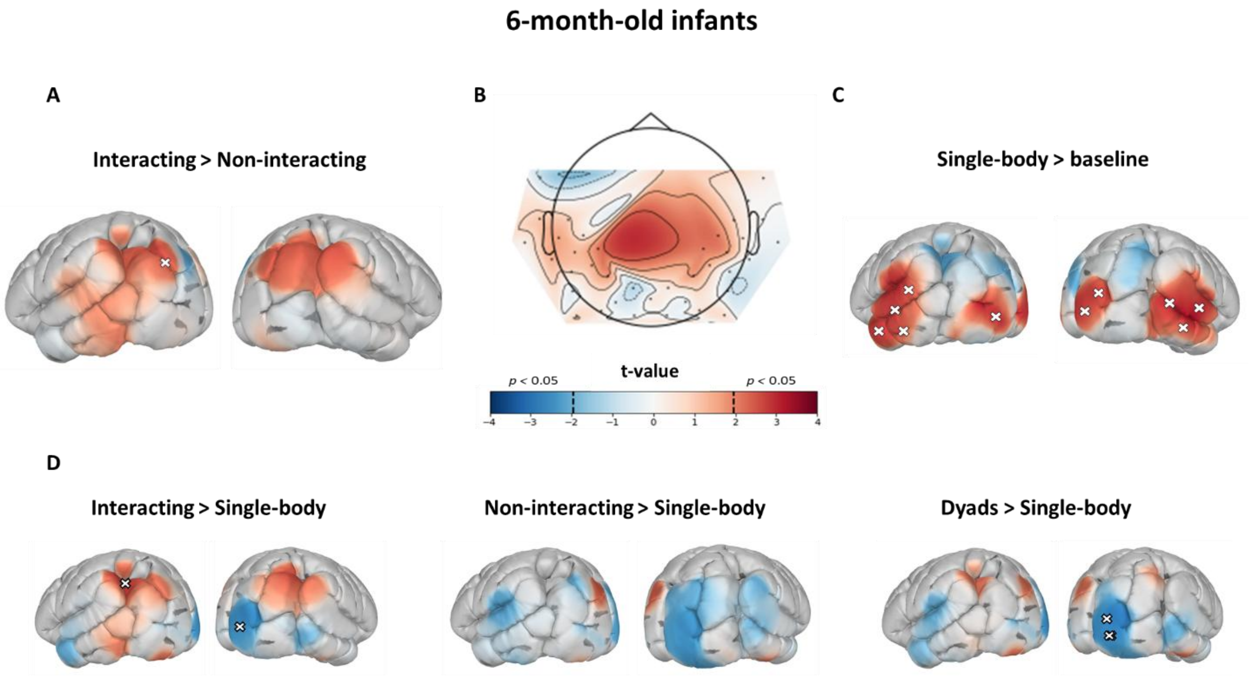

Figure S3: A. Distribution of the effect [interacting > non-interacting] in the channel-wise analysis in 6-month-olds. White Xs denote channels where the effect was significant. **B.** Results of the cluster-based permutation test in 6-month-olds: no cluster reached the significance level. **C.** Distribution of the effects [single-body motion > baseline] **D.** Distribution of the effect [interacting dyads > single-body], [non-interacting dyads > single-body] and [dyads > single-body] in 6-month-olds. White Xs denote the channel where effects were significant.

Ten-month-olds. The same analyses as above were carried out on fNIRS data collected in 10-month-olds. Results revealed a significant effect of social interaction (interacting – non-interacting difference above zero) in eight of the thirty-nine channels, located over occipital and temporoparietal areas (all ts > 1.96, all ps < 0.05) (Fig. S4A; Table S9). In one additional channel, located over the right middle occipital gyrus, the difference interacting – non-interacting was significantly below 0, indicating more activity for non-interacting dyads. The cluster-based permutation analysis revealed a significant cluster (p = 0.02) in the right hemisphere over an occipitoparietal and temporoparietal region (Fig. S4B).

The analysis modelling channel-wise activation for single-body motion perception revealed an effect in three channels, in the right inferior and middle temporal gyrus and in the right middle occipital gyrus (Fig. S4C). This effect of single-body motion did not overlap with the effect of dyad-perception (Fig. S4D). Modeling HbO concentration for the [interacting > single-body] contrast revealed an effect in nine channels (all ts > |1.96|, all p < 0.05). For eight of these nine channels, activity was higher for interacting dyads, while for one it was higher for single-body conditions. Modeling HbO concentration for the [non-interacting > single-body] contrast in 10-month-olds

revealed an effect in one channel, for which activity was higher for single-body conditions: POz-Oz (right cuneus/calcarine cortex), est. = -2.09 ± 0.72, $t_{(545.51)}$ = -2.92, p = 0.004. Lastly, modeling HbO concentration for the [dyads > single-body] contrast in 10-month-olds revealed an effect in three channels. P4-CP4 (right inferior parietal/angular cortex), showed higher activity for dyads (est. = 1.35 ± 0.64, $t_{(483.64)}$ = 2.15, p = 0.03); POz-Oz (right cuneus/calcarine cortex; est. = -1.29 ± 0.64, $t_{(483.64)}$ = -2, p = 0.04) and O1-Oz (left calcarine cortex; est. = -1.36 ± 0.64, $t_{(483.64)}$ = -2.11, p = 0.03), showed higher activity for single-body conditions.

In sum, the results of these analyses demonstrated an effect of social-interaction perception in posterior occipital/temporoparietal cortex, which was spatially dissociated from processing recruited for the perception of a single agent and its bodily motion. The effect of social interaction in 10-month-olds appears more widespread than the effect observed in 6-month-olds.

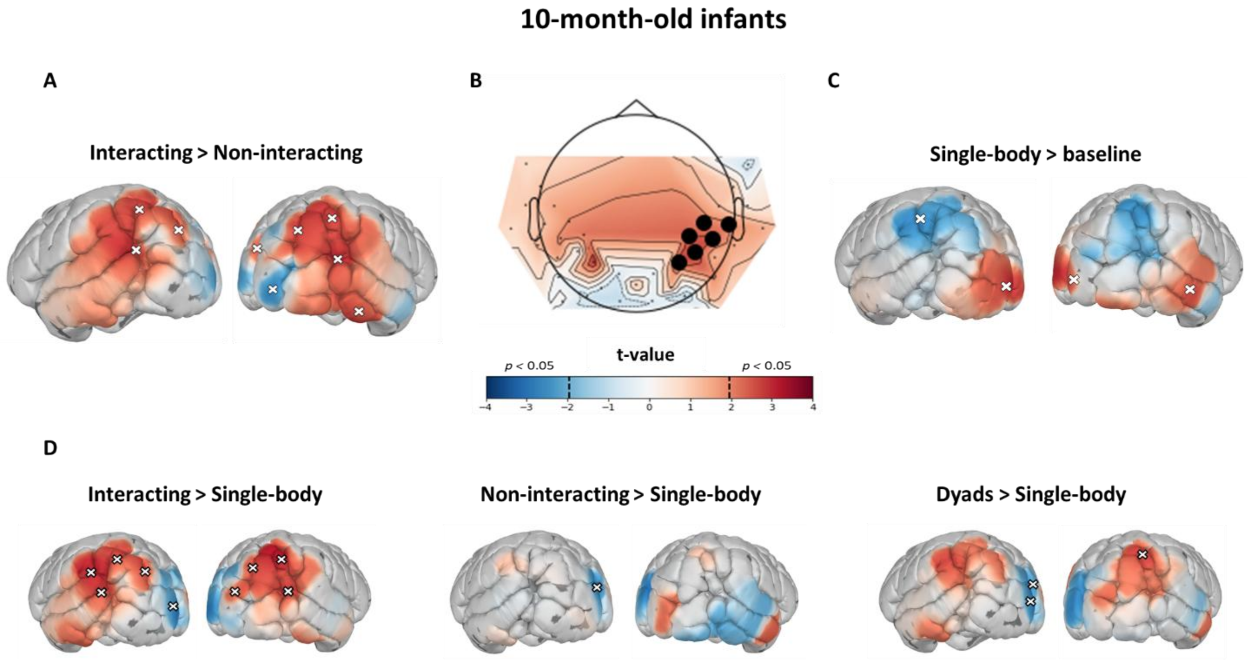

Figure S4: A. Distribution of the effect [interacting > non-interacting] in the channel-wise analysis in 10-month-olds. White Xs denote channels where the difference was significant. **B.** A cluster-based permutation test in 10-month-olds revealed one significant cluster (p = 0.02) covering occipitoparietal and temporoparietal regions (channels: P4-PO4, P4-P6, CP6-CP4, P4-CP4, CP6-P6, CP6-TP8). **C.** Distribution of the effect [single-body motion > baseline]. **D.** Distribution of the effect [interacting dyads > single-body], [non-interacting dyads > single-body] and [dyads > single-body] in 6 10-month-olds. White Xs denote channels where effects were significant.

Table S9: Channels showing a significant interacting – non-interacting effect in the channel-wise analysis in 6- and 10-month-old infants (Experiment 2).

**Channel**	**AAL**	**MNI coordinates (x, y, z)**	**Statistics**
**6-month-olds**			

P3-PO3	MOG (L) (51%)<br>ANG (L) (32%)	-36, -73, 34	*est.* = 1.55 ± 0.79, $t_{(533.66)}$ = 1.97, $p = 0.04$
**10-month-olds**			
POz-Oz	CUN (R) (41%)<br>CAL (R) (27%)	-3, -88, 22	*est.* = 1.6 ± 0.79, $t_{(533.66)}$ = 2.03, $p = 0.04$
O2-PO8	MOG (R) (88%)	30, -84, 7	*est.* = -1.78 ± 0.79, $t_{(533.66)}$ = -2.26, $p = 0.02$
P4-PO4	MOG (R) (61%)<br>ANG (R) (26%)	32, -75, 36	*est.* = 1.8 ± 0.79, $t_{(533.66)}$ = 2.29, $p = 0.02$
P4-CP4	IPG (R) (63%)<br>ANG (R) (32%)	44, -54, 47	*est.* = 1.71 ± 0.79, $t_{(533.66)}$ = 2.18, $p = 0.03$
CP6-P6	MTG (R) (37%)<br>STG (R) (31%)	55, -52, 22	*est.* = 1.88 ± 0.79, $t_{(533.66)}$ = 2.39, $p = 0.01$
TP10-TP8	ITG (R) (56%)<br>MTG (R) (36%)	57, -41, -18	*est.* = 1.69 ± 0.79, $t_{(533.66)}$ = 2.15, $p = 0.03$
P3-CP3	IPG (L) (51%)<br>ANG (L) (39%)	-49, -52, 44	*est.* = 1.6 ± 0.79, $t_{(533.66)}$ = 2.03, $p = 0.04$
P3-PO3	MOG (L) (51%)<br>ANG (L) (32%)	-36, -73, 34	*est.* = 1.55 ± 0.79, $t_{(533.66)}$ = 1.97, $p = 0.04$
CP5-P5	MTG (L) (42%)<br>STG (L) (34%)	-57, -49, 20	*est.* = 1.94 ± 0.79, $t_{(533.66)}$ = 2.47, $p = 0.01$

Note. CUN = cuneus; CAL = calcarine fissure and surrounding cortex; MOG = middle occipital gyrus; I/M/STG = inferior/middle/superior temporal gyrus; ANG = angular gyrus; IPG = inferior parietal gyrus; SMG = supramarginal gyrus. AAL labels represent probabilistic mapping of underlying cortical areas (specificity in parenthesis).

Model assumptions

6-month-old infants

Channel-wise response analysis. As for Experiment 1, we report the model specification formula (using *lmer*; Bates et al., 2015) for our channel-wise response analysis as well as our procedure for assessing model assumptions using DHARMA (Hartig, 2024) for Experiment 2.

Model specification:

```
channelwise_model = lmer(Difference ~ -1 + ch_name + (1|ID), data=data,
        REML=T,
        control=lmerControl(check.conv.singular = .makeCC(action = "ignore",  tol = 1e-4)))
```

Model assumptions:

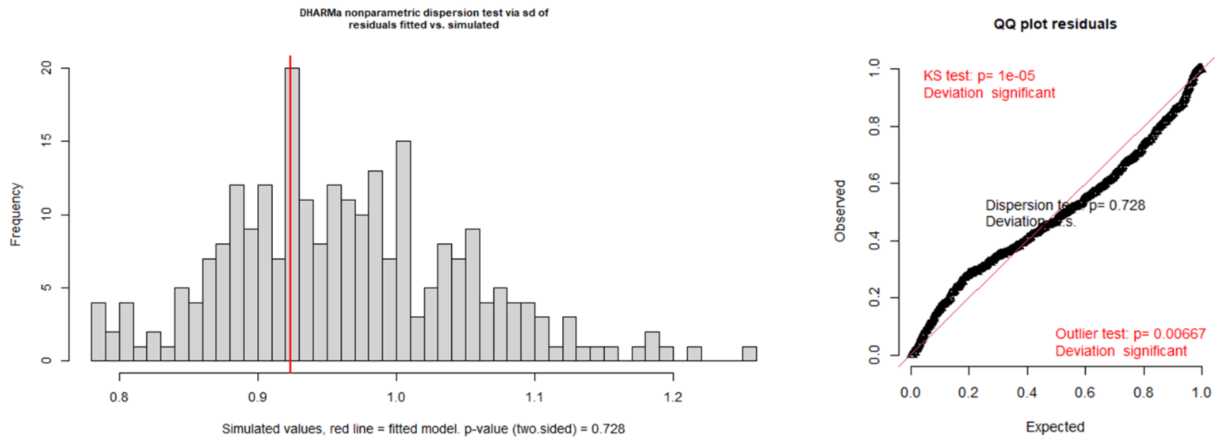

Figure S5: Model assumptions for the channel-wise response analysis in 6-month-old infants. Residuals dispersion was met (p = 0.73), while model residuals showed a deviation from normality (p < 0.001) and the presence of outliers (p = 0.007), possibly due to high number of data points. In fact, note that *"the p-value alone is not a good indicator of the extent to which your residuals deviate from assumptions. Specifically, if you have a lot of data points, residual diagnostics will nearly inevitably become significant, because having a perfectly fitting model is very unlikely. That, however, doesn't necessarily mean that you need to change your model"* (Hartig, 2024).

Because normality of residuals (p < 0.001) and presence of outliers (p = 0.007) were not met for this model, we re-run the model using robust mixed modeling (Koller, 2016).

Model specification:

robust_channelwise_model <- rlmer(Difference ~ -1 + ch_name + (1 | ID), data = data)

With this procedure, only one channel, P3-CP3 (left inferior parietal/ angular gyrus), was found significant for the [interacting > non-interacting] contrast (*est.* = 1.45, $t_{(296.95)}$ = 1.96, p = 0.05).

10-month-old infants

Channel-wise response analysis.

Model specification:

channelwise_model = lmer(Difference ~ -1 + ch_name + (1|ID), data=data,

REML=T,

control=lmerControl(check.conv.singular = .makeCC(action = "ignore", tol = 1e-4)))

Model assumptions:

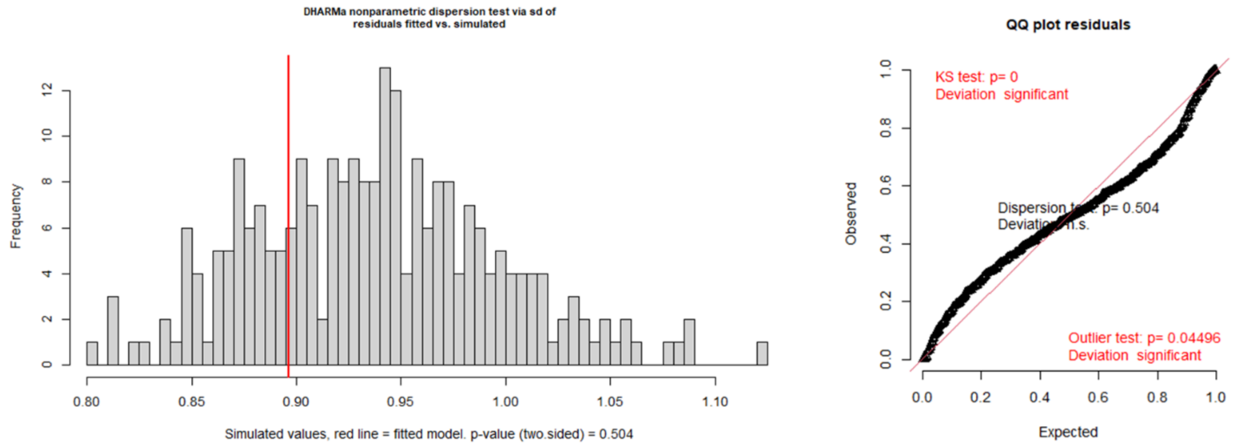

Figure S6: Model assumptions for the channel-wise response analysis in 10-month-olds. Residuals dispersion was met ($p = 0.5$), while model residuals showed a deviation from normality ($p < 0.001$) and the presence of outliers ($p = 0.04$), possibly due to high number of data points.

Because normality of residuals ($p < 0.001$) and presence of outliers ($p = 0.04$) were not met for this model, we re-run the model using robust mixed modeling (Koller, 2016).

Model specification:

robust_channelwise_model <- rlmer(Difference ~ -1 + ch_name + (1 | ID), data = data)

This procedure revealed seven channels that were significant for the [interacting > non-interacting] contrast using robust modeling. These are reported in Table S10. Six of these channels were also found with non-robust modeling and are formatted in bold.

Table S10: Significant channels for the Channel-wise response analysis – social facingness in pre-verbal infants using robust modeling.

Channel	Statistics	AAL2	Coordinates (x, y, z)
**P4-PO4**	***est.* = 1.57 ± 0.55, $t_{(533.7)}$ = 2.83, *p* = 0.004**	**MOG (R) (61%)<br>ANG (R) (26%)**	**32, -75, 36**
P4-P6	*est.* = 1.21 ± 0.55, $t_{(533.7)}$ = 2.18, *p* = 0.02	ANG (R) (78%)	45, -65, 32
**P4-CP4**	***est.* = 1.23 ± 0.55, $t_{(533.7)}$ = 2.22, *p* = 0.02**	**IPG (R) (63%)<br>ANG (R) (32%)**	**44, -54, 47**
**CP6-P6**	***est.* = 1.73 ± 0.55, $t_{(533.7)}$ = 3.12, *p* = 0.001**	**MTG (R) (37%)<br>STG (R) (31%)**	**55, -52, 22**
**P3-CP3**	***est.* = 1.2 ± 0.55, $t_{(533.7)}$ = 2.15, *p* = 0.03**	**IPG (L) (51%)<br>ANG (L) (39%)**	**-49, -52, 44**
**P3-PO3**	***est.* = 1.54 ± 0.55, $t_{(533.7)}$ = 2.76, *p* = 0.005**	**MOG (L) (51%)<br>ANG (L) (32%)**	**-36, -73, 34**

**CP5-P5**	***est.* = 1.31 ± 0.55, $t_{(533.7)}$ = 2.36, *p* = 0.01**	**MTG (L) (42%)<br>STG (L) (34%)**	**-57, -49, 20**

Note. MOG = middle occipital gyrus; M/STG = middle/superior temporal gyrus; ANG = angular gyrus; IPG = inferior parietal gyrus; SMG = supramarginal gyrus.

Analyses on HbR and HbT

As we only focused on HbO concentration in the main text, we report here the same analyses - channel-wise and ROI-based analyses- carried out on HbR and HbT in 6- and 10-month-old infants.

6-month-old infants

Channel-wise analysis. Modeling HbR for the [interacting > non-interacting] contrast in 6-month-olds revealed no significant channels. Modeling HbT for the [interacting > non-interacting] contrast revealed an effect in two channels, whereby HbT concentration was significantly higher for interaction perception – P3-PO3: left middle occipital/angular cortex; *est.* = 0.96 ± 0.48, $t_{(256.15)} = 2.01$, $p = 0.04$; PO10-PO8: right middle/inferior occipital cortex; ; *est.* = 0.95 ± 0.48, $t_{(256.15)} = 1.97$, p = 0.04.

10-month-old infants

Channel-wise analysis. Modeling HbR for the [interacting > non-interacting] contrast in 10-month-olds revealed an effect in seven channels, whereby differential HbR concentration was significantly lower for interacting dyads (Table S11). Modeling HbT for the [interacting > non-interacting] contrast revealed an effect in five channels. For four of these significant five, HbT concentration was significantly higher for interaction perception (Table S11).

Table S11: Significant channels for the Channel-wise response analysis on HbR and HbT concentration – social interaction contrast in 10-month-olds.

Channel	Statistics	AAL2	MNI coordinates (x, y, z)
**HbR**			
POz-Oz	*est.* = -0.91 ± 0.43, $t_{(526.88)}$ = -2.1, *p* = 0.03	CUN (R) (41%)<br>CAL (R) (27%)	-3, -88, 22
P4-PO4	*est.* = -1.11 ± 0.43, $t_{(526.88)}$ = -2.54, *p* = 0.01	MOG (R) (61%)<br>ANG (R) (26%)	32, -75, 36
CP6-P6	*est.* = -0.94 ± 0.43, $t_{(526.88)}$ = -2.16, *p* = 0.03	MTG (R) (37%)<br>STG (R) (31%)	55, -52, 22
TP10-TP8	*est.* = -0.93 ± 0.43, $t_{(526.88)}$ = -2.15, *p* = 0.03	ITG (R) (56%)<br>MTG (R) (36%)	57, -41, -18
P3-CP3	*est.* = -0.91 ± 0.43, $t_{(526.88)}$ = -2.1, *p* = 0.03	IPG (L) (51%)<br>ANG (L) (39%)	-49, -52, 44
CP5-P5	*est.* = -1.01 ± 0.43, $t_{(526.88)}$ = -2.32, *p* = 0.02	MTG (L) (42%)<br>STG (L) (34%)	-57, -49, 20
CP5-CP3	*est.* = -0.92 ± 0.43, $t_{(526.88)}$ = -2.14, *p* = 0.03	SMG (L) (58%)<br>IPG (L) (22%)	-58, -39, 33
**HbT**			

O2-PO8	*est.* = -1.07 ± 0.39, $t_{(594.53)}$ = -2.76, *p* = 0.006	MOG (R) (88%)	30, -84, 7
P4-CP4	*est.* = 0.92 ± 0.39, $t_{(594.53)}$ = 2.37, *p* = 0.01	IPG (R) (63%)<br>ANG (R) (32%)	44, -54, 47
CP6-P6	*est.* = 0.94 ± 0.39, $t_{(594.53)}$ = 2.43, *p* = 0.01	MTG (R) (37%)<br>STG (R) (31%)	55, -52, 22
P3-PO3	*est.* = 0.78 ± 0.39, $t_{(594.53)}$ = 2.01, *p* = 0.04	MOG (L) (51%)<br>ANG (L) (32%)	-36, -73, 34
CP5-P5	*est.* = 0.93 ± 0.39, $t_{(594.53)}$ = 2.41, *p* = 0.01	MTG (L) (42%)<br>STG (L) (34%)	57, -49, 20

Note. MOG = middle occipital gyrus; CAL = calcarine fissure and surrounding cortex; CUN = cuneus; I/M/STG = inferior/middle/superior temporal gyrus; ANG = angular gyrus; IPG = inferior parietal gyrus; SMG = supramarginal gyrus.

Evaluation of the hemodynamic response peak

In order to choose a sensitive time window for our analyses, we performed a peak detection procedure. This showed that, in all groups, the average hemodynamic response (across all channels) peaked at around 8 seconds (Fig. S7) – which is in contrast with the canonical behavior of the hemodynamic response, peaking around 5/6 seconds (Poldrack et al., 2024; but see Schneider et al., 2014, for similar fNIRS results). To account for this slight delay in the average neural response, we selected an analysis window of [0, 14] seconds (see main text).

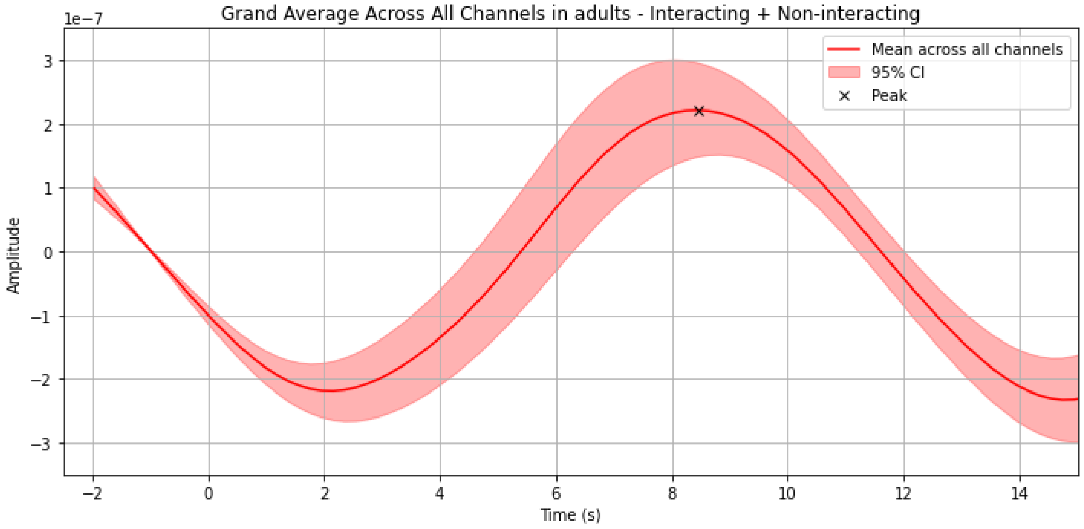

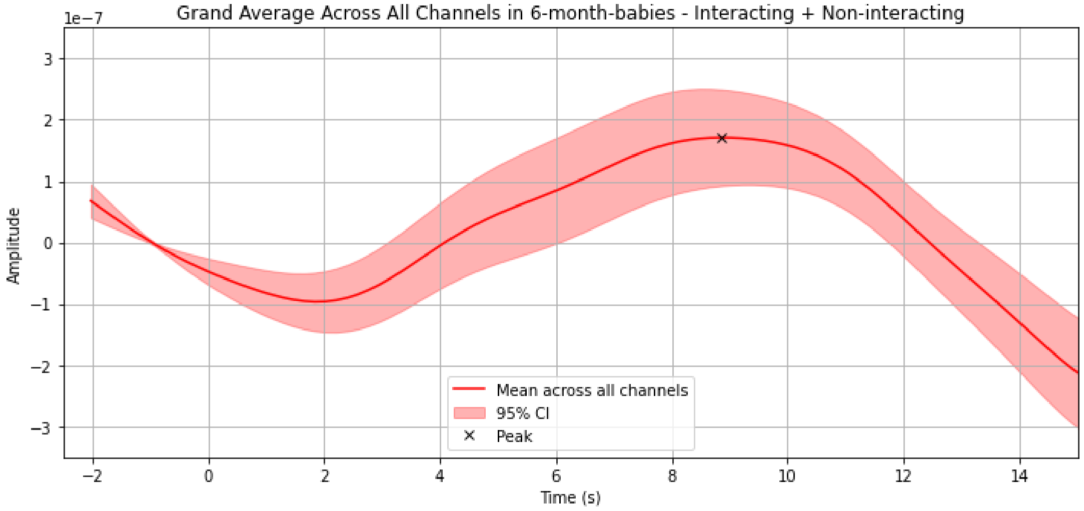

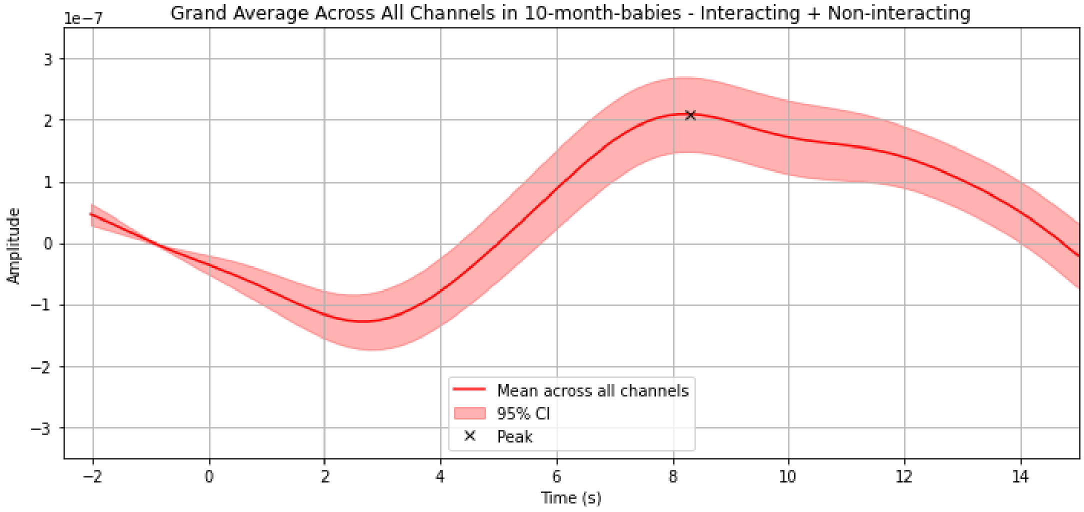

Figure S7: From top to bottom: HbO response peak (across channels) for both conditions averaged (interacting and non-interacting dyads) in adults – peak = 8.45 sec; 6-month-olds – peak = 8.85 sec; and 10-month-olds – peak = 8.29 sec.

Controlling for the distance between the two bodies in our stimuli

Experiment 1 (adults) involved stimuli used in a previous fMRI study (Bellot et al., 2021) and consisting of 2-sec video-clips of PLDs depicting two human bodies facing and moving toward or away from each other. Thirteen animations of individual bodies performing various familiar movements were taken from the Communicative Interactions Database (CID; Manera et al., 2010). Animations were trimmed so to have all the same duration of 2 sec, and were paired to form 9 interacting dyads, by positioning each pair face-to-face. Non-interacting dyads were created by horizontally flipping the individual bodies in each interacting dyad, yielding the same number of back-to-back dyads. In the two conditions, the two bodies were at matched distance from each other such that interpersonal orientation was the only feature that differed between interacting and non-

interacting dyads (see main text). Briefly, we selected the frames in which the 2 bodies were the farthest (F1) and the closest (F2). In each selected frame, the most informative points of each body (i.e., shoulders, hands, hips, ankles) were used to compute the polygon centroid point of the body. The distance between the 2 bodies in a movie was quantified as the number of pixels along the x-axis between the centroids of the 2 bodies at F1 and F2 (i.e., D1 for F1 and D2 for F2): $D = |D1-D2|$. Statistical analysis (two-tailed t-test) showed that distance D between the 2 bodies was comparable for interacting and non-facing dyads.

Stimuli for Experiment 2 (infants) were similar to the first set of Experiment 1, except that 9 animations of individual bodies taken from the CID (Manera et al., 2010) were trimmed so that each lasted 5 sec (instead of 2 sec). By pairing individual bodies, eight different facing/interacting and corresponding non-facing/non-interacting dyads were created. The distance between bodies in the dyads was matched between interacting and non-interacting stimuli as done in Experiment 1 (see main text). Moreover, as an additional sanity check on the stimuli, we calculated the average distance between the two bodies across frames (mean_D). Each facing and non-facing video had its own mean_D value, which we compared using a paired-samples t-test. The mean distance in pixels between the two bodies in facing and non-facing dyads videos was comparable and was thus controlled for ($mean\_D_{facing} = 789 \pm 42$, $mean\_D_{non\text{-}facing} = 795 \pm 47$; $t_{(7)} = -0.33$, $p = 0.75$).

Looking time analyses

The final set of an analyses was carried out to assess the relationship between pSTS activity and attention. Since pSTS is part of the ventral attention network (Corbetta & Schulman, 2002; Vossel et al., 2014), we asked to what extent the pSTS response to our stimuli reflected the variance in the attention to the different types of stimuli. To test this, we measured the looking times toward the stimulus, for each trial, based on the analysis of the videos of infants (n = 38; 3 videos corresponding to 3 10-month-old infants were not included due to a very low quality), recorded during the fNIRS experimental session. This analysis was implemented in the following steps:

1) a researcher (M.M.) decomposed each video into individual frames (down-sampled from 24 fps to 6 fps);

2) M.M. categorized experimental trial frames as either ON or OFF, depending on whether the infant was judged to be looking at the trial or not, respectively.

3) another author (E. S.) repeated step 2 for a sample corresponding to the 10% of the videos (n=4), in order to calculate inter-rater agreement (Cohen's kappa, k). For all 4 videos, the inter-rater agreement was very high (k = 0.95 (98%), k = 0.88 (96%), k = 0.79 (97%), and k = 0.9 (97%);

4) for each participant and each trial, the proportion of ON frames out of the total number of frames was calculated and used to perform statistical assessing (i) to what extent the mean looking times were modulated by conditions and age groups (Fig. S8), and (ii) whether neural responses in pSTS were predicted by the infants' looking behavior.

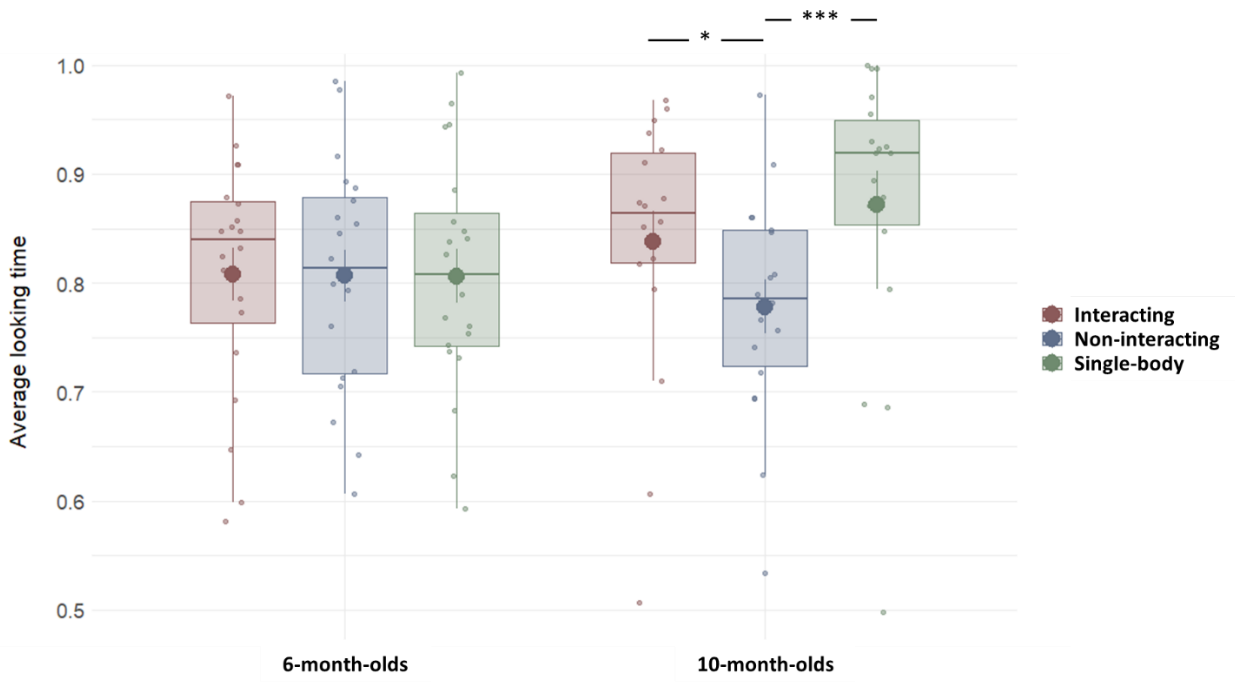

Figure S8. **Results of the looking time analyses**. The ANOVA with Age (6, 10 months) as between-subjects factor and Condition (interacting, non-interacting, single) as within-subjects factor, revealed a main effect of Condition, $F_{(2,72)} = 3.58$, $p = 0.03$, but not an effect of Age, $F_{(1,36)} = 0.51$, $p = 0.48$, and a significant interaction between the two factors, $F_{(2,72)} = 4$, $p = 0.02$. Pairwise comparisons (Tukey-corrected) showed that 10-month-olds looked more at interacting dyads compared to non-interacting dyads (est. = 0.06 ± 0.02, $t = 2.44$, $p = 0.04$) and more to single-body motion than to non-interacting dyads (est. = 0.09 ± 0.02, $t = 3.85$, $p < 0.001$). No differences between conditions were found for the younger age group (all other comparisons: ts < 1.42, ps > 0.34).

Covariate models. In the main text, we report models in which by-condition, centered looking times were added as a covariate predicting the response in left and right pSTS. Here, we integrate this analysis with models run 1) on single-body motion for all infants and 2) on the two age groups separately, for the [Interacting – Non-interacting] contrast and for single-body motion.

1) Linear regression was run with Looking times as a predictor of left and right pSTS activity for single-body motion. We found no evidence of an association between looking times for single-body motion and left (est. = 1.33 ± 2.37, $t = 0.56$, $p = 0.58$) or right (est. = -4.08 ± 2.86, $t = -1.43$, $p = 0.16$) pSTS activation.

2) Linear mixed regressions were run with Condition (Interacting / Non-interacting) as fixed effect and Looking times as a covariate predicting the response in left and right pSTS. In 6-month-olds we found no significant effect for left pSTS (Condition: $F_{(1,18)} = 1.3$, $p = 0.27$; Looking times: $F_{(1,30)} = 0.01$, $p = 0.91$; interaction: $F_{(1,26)} = 0.43$, $p = 0.52$) or right pSTS (Condition: $F_{(1,18)} = 2.66$, $p = 0.12$; Looking times: $F_{(1,32)} = 0.07$, $p = 0.79$; interaction: $F_{(1,25)} = 0.04$, $p = 0.83$). In 10-month-olds, we found a Condition effect ($F_{(1,18)} = 5.06$, $p = 0.03$), but no modulation of Looking times ($F_{(1,25)}$

= 2.04, p = 0.16), or interaction between the two ($F_{(1,23)}$ = 0.13, p = 0.72) for the left pSTS and the same results for the right pSTS (Condition: $F_{(1,32)}$ = 7.27, p = 0.01; Looking times: $F_{(1,32)}$ = 2.18, p = 0.15; interaction: $F_{(1,32)}$ = 0.31, p = 0.58) (note that, for this last model, the model fit was singular, and thus results might not be reliable).

Linear regressions linking pSTS response to single-body motion to looking times for this condition were also run in the two age groups separately. We found no evidence of this association in neither of the two infant groups (6-months: est. = -3.08 ± 5.15, t = -0.6, p = 0.56; 10-months: est. = -3.21 ± 3.29, t = -0.97, p = 0.34).

Bayesian hypothesis testing

Bayesian statistics was utilized to provide further evidence in favor, or against, of our tested hypotheses (Van de Schoot et al., 2021).

For Experiment 1 (adults), we implemented a Bayesian linear mixed model (with *brms*; Bürkner, 2017) replicating the channel-wise analysis in the main text. Bayes factors were computed, for each channel parameter, as BF_{10} to assess evidence in favor of the alternative hypothesis (H_1; contrast [Interacting - Non-interacting] > 0) over the null hypothesis (H_0), based on prior and posterior samples of a single parameter (using *bayestestR*; Makowski et al., 2019). We specified an informative prior for the regression coefficients, reflecting theoretical expectations of a positive effect in adults, while allowing for uncertainty and the possibility of negative effects. The results are shown in Table S13.

Table S12: Bayes factors calculated for Experiment 1 (adults) on the social interaction effect for each channel. Est. = estimate, CIs = credible intervals, BF = Bayes factor.

Channel	Statistics (est.[95%CIs])	Channel	Statistics (est.[95%CIs])
POO2-PPO2h	*est* .= -0.07[-0.63,0.5], BF_{10} = 0.59	TPP10h-PPO10h	*est* .= 0.1[-0.47,0.68], BF_{10} = 0.6
POO2-POO1	*est* .= 0.24[-0.24,0.83], BF_{10} = 0.8	TPP10h-TPP8h	*est* .= 0.3[-0.24,0.85], BF_{10} = 0.93
POO2-OI2h	*est* .= 0.14[-0.42,0.72], BF_{10} = 0.54	CPP6h-PPO6h	*est* .= 0.55[-0.03,1,16], BF_{10} = 3.37
CPP3h-CPP5h	*est* .= 0.32[-0.26,0.9], BF_{10} = 1.03	CPP6h-TPP8h	*est* .= 0.44[-0.13,0.99], BF_{10} = 1.55
CPP3h-CCP3h	*est* .= 0.28[-0.3,0.84], BF_{10} = 0.88	CPP6h-CPP4h	*est* .= 0.5[-0.11,1.07], BF_{10} = 2.42
PPO5h-CPP5h	*est* .= 0.59 [0.03,1.15], BF_{10} = 4.91	CPP6h-CCP6h	*est* .= 0.39[-0.2,1.01], BF_{10} = 1.27
PPO9h-POO9h	*est* .= 0.27[-0.28,0.87], BF_{10} = 0.79	CCP4h-CPP4h	*est* .= 0.48[-0.11,1.08], BF_{10} = 1.99
PPO9h-TPP9h	*est* .= 0.08[-0.5,0.66], BF_{10} = 0.53	CCP4h-CCP6h	*est* .= 0.62[-0.04,1.20], BF_{10} = 4.84
TPP7h-CPP5h	*est* .= 0.61[0.06,1.19], BF_{10} = 6.05	TTP8h-TPP8h	*est* .= 0.4[-0.19,0.99], BF_{10} = 1.32
TPP7h-TPP9h	*est* .= 0.42[-0.16,0.99], BF_{10} = 1.48	TTP8h-CCP6h	*est* .= 0.31[-0.26,0.87], BF_{10} = 0.96
TPP7h-TTP7h	*est* .= 0.32[-0.25,0.89], BF_{10} = 0.96	TTP8h-FTT8h	*est* .= 0.22[-0.34,0.83], BF_{10} = 0.66
CCP5h-CPP5h	*est* .= 0.59[0.01,1.2], BF_{10} = 3.88	FTT10h-FTT8h	*est* .= -0.32[-0.92,0.29], BF_{10} = 1.08
CCP5h-TTP7h	*est* .= 0.2[-0.37,0.77], BF_{10} = 0.7	FTT10h-FFT10h	*est* .= -0.18 [-0.8,0.4], BF_{10} = 0.65

CCP5h-CCP3h	*est* .= 0.34[-0.23,0.92], BF_{10} = 1.03	OI1h-POO1	*est* .= 0.29[-0.28,0.87], BF_{10} = 0.84
FTT7h-TTP7h	*est* .= 0.26[-0.31,0.81], BF_{10} = 0.77	OI1h-POO9h	*est* .= 0.24[-0.32,0.78], BF_{10} = 0.79
FTT7h-FTT9h	*est* .= -0.14[-0.72,0.43], BF_{10} = 0.63	OI1h-OI2h	*est* .= 0.1[-0.45,0.68], BF_{10} = 0.49
FFT9h-FTT9h	*est* .= -0.31[-0.9,0.28], BF_{10} = 0.96	PPO1h-PPO2h	*est* .= 0.09[-0.46,0.65], BF_{10} = 0.55
POO10h-PPO10h	*est* .= 0.58[0.03,1.15], BF_{10} = 4.39	PPO1h-POO1	*est* .= 0.12[-0.45,0.7], BF_{10} = 0.61
POO10h-OI2h	*est* .= 0.1[-0.47,0.68], BF_{10} = 0.58		

Regarding Experiment 2 (infants), for omnibus effects related to the factorial model Channel*Age in the main analyses, a hierarchical model comparison approach was performed using Bayesian linear mixed models (with *brms*; Bürkner, 2017). For infants, we used a weakly informative prior centered on no effect (zero), reflecting no strong prior belief about the direction of the effect. First, a model with only channel parameters was compared to an intercept-only model. This showed a better performance of the former (BF_{10} = 13.47). Adding the Age factor to the channel model did not explain additional variance (BF_{10} = 0.82), as did not adding the interaction term (BF_{10} = 0.13).

Like for adults, for each channel parameter, Bayes factors were computed as BF_{10} to assess evidence in favor of the alternative hypothesis (H_1; contrast [Interacting - Non-interacting] ≠ 0) over the null hypothesis (H_0), and based on prior and posterior samples of a single parameter (using *bayestestR*; Makowski et al., 2019). The results are reported in Table S13.

Table S13. Bayes factors calculated for Experiment 2 (infants) on the social interaction effect for each channel. Est. = estimate, CIs = credible intervals, BF = Bayes factor.

Channel	Statistics (est.[95%CIs])	Channel	Statistics (est.[95%CIs])
POz-Oz	*est* .= 0.23[-0.5,0.98], BF_{10} = 0.89	O2-PO8	*est* .= -0.51[-1.23,0.22], BF_{10} = 1.98
POz-PO4	*est* .= -0.22[-0.94,0.54], BF_{10} = 0.89	PO10-PO8	*est* .= 0.14[-0.62,0.88], BF_{10} = 0.77
POz-PO3	*est* .= -0.57[-1.26,0.11], BF_{10} = 2.52	P4-PO4	*est* .= 0.58[-0.14,1.32], BF_{10} = 2.31
PO9-PO7	*est* .= -0.08[-0.82,0.7], BF_{10} = 0.74	P4-P6	*est* .= 0.35[-0.37,1.07], BF_{10} = 1.07
P7-PO7	*est* .= -0.27[-0.99,0.48], BF_{10} = 1	P4-CP4	*est* .= 0.54[-0.18,1.26], BF_{10} = 2.39
P7-P5	*est* .= 0.16[-0.58,0.87], BF_{10} = 0.8	P8-PO8	*est* .= -0.08[-0.8,0.64], BF_{10} = 0.8
P7-TP7	*est* .= 0.15[-0.54,0.82], BF_{10} = 0.73	P8-P6	*est* .= -0.17[-0.9,0.55], BF_{10} = 0.84
P3-P5	*est* .= -0.08[-0.8,0.63], BF_{10} = 0.83	P8-TP8	*est* .= 0.06[-0.73,0.84], BF_{10} = 0.78
P3-CP3	*est* .= 0.52[-0.21,1.23], BF_{10} = 2.01	CP6-P6	*est* .= 0.48[-0.28,1.21], BF_{10} = 1.54
P3-PO3	*est* .= 0.58[-0.16,1.31], BF_{10} = 2.71	CP6-CP4	*est* .= 0.46[-0.27,1.22], BF_{10} = 1.89
CP5-P5	*est* .= 0.45[-0.32,1.22], BF_{10} = 1.68	CP6-TP8	*est* .= 0.01[-0.71,0.75], BF_{10} = 0.7
CP5-TP7	*est* .= 0.17[-0.6,0.94], BF_{10} = 0.87	CP6-C6	*est* .= 0.09[-0.68,0.81], BF_{10} = 0.75
CP5-CP3	*est* .= 0.2[-0.55,0.92], BF_{10} = 0.87	TP10-TP8	*est* .= 0.09[-0.64,0.86], BF_{10} = 0.75
CP5-C5	*est* .= 0.18[-0.56,0.93], BF_{10} = 0.81	T8-TP8	*est* .= -0.13[-0.88,0.61], BF_{10} = 0.79
TP9-TP7	*est* .= 0.12[-0.57,0.84], BF_{10} = 0.77	T8-C6	*est* .= -0.09[-0.84,0.61], BF_{10} = 0.79

T7-TP7	*est* .= 0.1[-0.64,0.84], BF_{10} = 0.78	T8-FT8	*est* .= -0.3[-1.05,0.46], BF_{10} = 1.05
T7-C5	*est* .= -0.07[-0.78,0.62], BF_{10} = 0.75	O1-Oz	*est* .= -0.52[-1.24,0.19], BF_{10} = 2.08
T7-FT7	*est* .= -0.19[-0.94,0.55], BF_{10} = 0.87	O1-PO7	*est* .= -0.27[-0.98,0.46], BF_{10} = 0.95
O2-Oz	*est* .= -0.3[-1.06,0.47], BF_{10} = 1.03	O1-PO3	*est* .= -0.38[-1.12,0.39], BF_{10} = 1.23
O2-PO4	*est* .= 0.02[-0.71,0.79], BF_{10} = 0.76		

This same analysis was also carried out for the contrast [Single-body – baseline]. The results are reported in Table S14.

Table S14. Bayes factors calculated for Experiment 2 (infants) on the single-body motion effect for each channel. Est. = estimate, CIs = credible intervals, BF = Bayes factor.

Channel	Statistics (est.[95%CIs])	Channel	Statistics (est.[95%CIs])
POz-Oz	*est* .= -0.27[-0.8,0.29], BF_{10} = 0.81	O2-PO8	*est* .= 0.15[-0.4,0.71], BF_{10} = 0.63
POz-PO4	*est* .= -0.05[-0.57,0.49], BF_{10} = 0.61	PO10-PO8	*est* .= 0.11[-0.44,0.65], BF_{10} = 0.64
POz-PO3	*est* .= -0.13[-0.69,0.45], BF_{10} = 0.61	P4-PO4	*est* .= -0.21[-0.75,0.35], BF_{10} = 0.83
PO9-PO7	*est* .= -0.1[-0.65,0.43], BF_{10} = 0.67	P4-P6	*est* .= -0.44[-0.97,0.11], BF_{10} = 1.86
P7-PO7	*est* .= 0.26[-0.26,0.81], BF_{10} = 0.83	P4-CP4	*est* .= -0. 6[-1.12,-0.05], BF_{10} = 6.77
P7-P5	*est* .= -0.17[-0.73,0.39], BF_{10} = 0.74	P8-PO8	*est* .= -0.24[-0.81,0.30], BF_{10} = 0.79
P7-TP7	*est* .= -0.2 [-0.76,0.36], BF_{10} = 0.72	P8-P6	*est* .= -0.15[-0.72,0.4], BF_{10} = 0.74
P3-P5	*est* .= -0.25[-0.82,0.32], BF_{10} = 0.87	P8-TP8	*est* .= 0.33[-0.2,0.84], BF_{10} = 1.16
P3-CP3	*est* .= -0.58[-0.1,-0.04], BF_{10} = 5.82	CP6-P6	*est* .= -0.22[-0.8,0.34], BF_{10} = 0.7
P3-PO3	*est* .= -0.38[-0.93,0.16], BF_{10} = 1.46	CP6-CP4	*est* .= -0.15[-0.7,0.41], BF_{10} = 0.62
CP5-P5	*est* .= -0.25[-0.77,0.31], BF_{10} = 0.9	CP6-TP8	*est* .= 0.47[-0.07,1.02], BF_{10} = 2.44
CP5-TP7	*est* .= 0.2[-0.34,0.74], BF_{10} = 0.63	CP6-C6	*est* .= 0.35[-0.2,0.9], BF_{10} = 1.15
CP5-CP3	*est* .= -0.29[-0.87,0.28], BF_{10} = 1.03	TP10-TP8	*est* .= -0.12[-0.66,0.44], BF_{10} = 0.61
CP5-C5	*est* .= 0.23[-0.30,0.78], BF_{10} = 0.85	T8-TP8	*est* .= 0.94[0.4,1.5], BF_{10} = 109.16
TP9-TP7	*est* .= -0.14[-0.69,0.44], BF_{10} = 0.8	T8-C6	*est* .= 0.52[-0.05,1.07], BF_{10} = 3.19
T7-TP7	*est* .= 0.37[-0.17,0.91], BF_{10} = 1.31	T8-FT8	*est* .= -0.09[-0.63,0.44], BF_{10} = 0.61
T7-C5	*est* .= 0.32[-0.22,0.88], BF_{10} = 0.97	O1-Oz	*est* .= 0.49[-0.05,1.04], BF_{10} = 2.82
T7-FT7	*est* .= 0.36[-0.2,0.9], BF_{10} = 1.27	O1-PO7	*est* .= 0.73 [0.15,1.31], BF_{10} = 13.49
O2-Oz	*est* .= 0.69 [0.27,1.22], BF_{10} = 13.39	O1-PO3	*est* .= 0.34[-0.22,0.9], BF_{10} = 1.37
O2-PO4	*est* .= 0.03[-0.52,0.57], BF_{10} = 0.52		

Specifics on the motion artifacts correction technique implemented in our work

The preprocessing of the fNIRS signal in our work included, before converting to hemoglobin concentration, treating optical density (OD) data by applying temporal-derivative distribution repair (TDDR; Fishburn et al., 2019), an artifact correction procedure based on robust regression, which removes baseline shift and spike artifacts, and motion artifacts correction using a custom-made wavelet-based filtering function (Molavi & Dumont, 2012; Brigadoi et al., 2014), used to further remove large-amplitude spike artifacts in the data (see this article's open source materials for the custom-made code). In this last step, motion artifacts were removed using a wavelet-based filtering method. Signals were decomposed with a Daubechies-5 wavelet (db5), and outlier wavelet coefficients were identified using an interquartile range criterion and suppressed prior to signal reconstruction. A conservative threshold (α = 5) was used to balance artifact attenuation and signal distortion (Fig. S9).

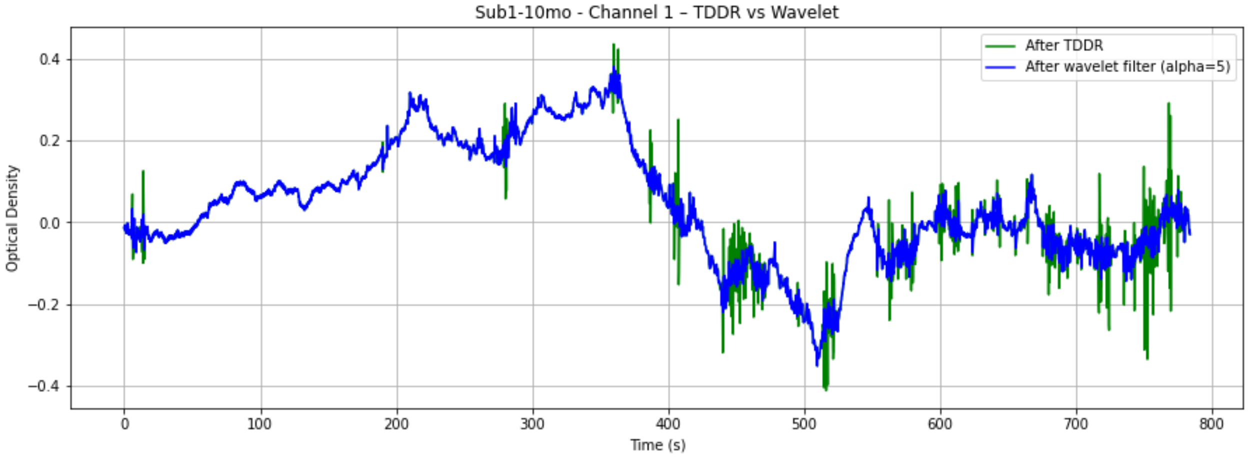

Figure S9: Pre-processed OD signal after TDDR (in green) and after TDDR + wavelet-based filtering (in blue). The latter was effective in removing large-amplitude spikes that still contaminated the data.

References

Bates, D., Mächler, M., Bolker, B., & Walker, S. (2015). Fitting linear mixed-effects models using lme4. *Journal of Statistical Software*, *67*, 1-48.

Bellot, E., Abassi, E., & Papeo, L. (2021). Moving toward versus away from another: how body motion direction changes the representation of bodies and actions in the visual cortex. *Cerebral Cortex*, *31*(5), 2670-2685.

Corbetta, M., & Shulman, G. L. (2002). Control of goal-directed and stimulus-driven attention in the brain. *Nature Reviews Neuroscience*, *3*(3), 201-215.

Fu, X., & Richards, J. E. (2021). devfOLD: a toolbox for designing age-specific fNIRS channel placement. Neurophotonics, 8(4), 045003-045003.

Hartig F. 2024. DHARMa: residual diagnostics for hierarchical (multi-level/mixed) regression models. R package version 0.4.7. https://CRAN.R-project.org/package=DHARMa.

Koller, M. (2016). robustlmm: an R package for robust estimation of linear mixed-effects models. *Journal of Statistical Software*, *75*, 1-24.

Manera, V., Schouten, B., Becchio, C., Bara, B. G., & Verfaillie, K. (2010). Inferring intentions from biological motion: a stimulus set of point-light communicative interactions. *Behavior Research Methods*, *42*(1), 168-178.

Poldrack, R. A., Mumford, J. A., & Nichols, T. E. (2024). *Handbook of functional MRI data analysis*. Cambridge University Press.

Schneider, S., Christensen, A., Häußinger, F. B., Fallgatter, A. J., Giese, M. A., & Ehlis, A. C. (2014). Show me how you walk and I tell you how you feel—a functional near-infrared spectroscopy study on emotion perception based on human gait. *NeuroImage*, *85*, 380-390.

Van de Schoot, R., Depaoli, S., King, R., Kramer, B., Märtens, K., Tadesse, M. G., ... & Yau, C. (2021). Bayesian statistics and modelling. *Nature Reviews Methods Primers*, *1*(1), 1.

Vossel, S., Geng, J. J., & Fink, G. R. (2014). Dorsal and ventral attention systems: distinct neural circuits but collaborative roles. *The Neuroscientist*, *20*(2), 150-159.

Zimeo Morais, G. A., Balardin, J. B., & Sato, J. R. (2018). fNIRS Optodes' Location Decider (fOLD): a toolbox for probe arrangement guided by brain regions-of-interest. *Scientific Reports*, *8*(1), 3341.
